# Metabolic modeling of *Halomonas campaniensis* improves polyhydroxybutyrate production under nitrogen limitation

**DOI:** 10.1007/s00253-024-13111-8

**Published:** 2024-04-25

**Authors:** Carolina Deantas-Jahn, Sebastián N. Mendoza, Cuauhtemoc Licona-Cassani, Camila Orellana, Pedro A. Saa

**Affiliations:** 1 Departamento de Ingeniería Química y Bioprocesos, Escuela de Ingeniería, Pontificia Universidad Católica de Chile, Santiago, Chile; 2 Systems Biology Lab, Vrije Universiteit Amsterdam, Amsterdam, Netherlands; 3 Núcleo de Innovación de Sistemas Biológicos (NISB), FEMSA Biotechnology Center, Tecnológico de Monterrey, Monterrey, Mexico; 4 Escuela de Ingeniería y Ciencias, Tecnológico de Monterrey, Monterrey, Mexico; 5 Instituto de Ingeniería Matemática y Computacional, Pontificia Universidad Católica de Chile, Santiago, Chile

**Keywords:** Genome-scale metabolic model, Flux balance analysis, *Halomonas*, Polyhydroxyalkanoate, Extremophile

## Abstract

**Abstract:**

Poly-hydroxybutyrate (PHB) is an environmentally friendly alternative for conventional fossil fuel-based plastics that is produced by various microorganisms. Large-scale PHB production is challenging due to the comparatively higher biomanufacturing costs. A PHB overproducer is the haloalkaliphilic bacterium *Halomonas campaniensis*, which has low nutritional requirements and can grow in cultures with high salt concentrations, rendering it resistant to contamination. Despite its virtues, the metabolic capabilities of *H. campaniensis* as well as the limitations hindering higher PHB production remain poorly studied. To address this limitation, we present HaloGEM, the first high-quality genome-scale metabolic network reconstruction, which encompasses 888 genes, 1528 reactions (1257 gene-associated), and 1274 metabolites. HaloGEM not only displays excellent agreement with previous growth data and experiments from this study, but it also revealed nitrogen as a limiting nutrient when growing aerobically under high salt concentrations using glucose as carbon source. Among different nitrogen source mixtures for optimal growth, HaloGEM predicted glutamate and arginine as a promising mixture producing increases of 54.2% and 153.4% in the biomass yield and PHB titer, respectively. Furthermore, the model was used to predict genetic interventions for increasing PHB yield, which were consistent with the rationale of previously reported strategies. Overall, the presented reconstruction advances our understanding of the metabolic capabilities of *H. campaniensis* for rationally engineering this next-generation industrial biotechnology platform.

**Key points:**

- *A comprehensive genome-scale metabolic reconstruction of H. campaniensis was developed.*
- *Experiments and simulations predict N limitation in minimal media under aerobiosis.*
- *In silico media design increased experimental biomass yield and PHB titer.*

**Supplementary Information:**

The online version contains supplementary material available at 10.1007/s00253-024-13111-8.

## Introduction

*Halomonas* is a genus that comprises gram-negative halophilic/halotolerant aerobic bacteria, which can thrive at alkali pH in relatively high salt (NaCl) concentrations (Romano et al. 2005). These harsh culture conditions prevent contamination by other microbial strains (Tan et al. 2011). Open and unsterile cultivation of *Halomonas* spp. under such conditions is not only technically feasible, but also economically attractive (e.g., avoids expensive sterilization steps) (Ling et al. 2019) and environmentally friendly (e.g., seawater may be used for growth media solvent instead of fresh water) (Yue et al. 2014). Unlike extreme halophilic archaea that require extreme salt concentrations (25–30% w/v), *Halomonas* spp. grow in moderate-to-high salt conditions (3–15% w/v), thereby reducing growth medium requirements and cost, and avoiding accelerated corrosion of common stainless steel equipment like fermentors (Quillaguamán et al. 2005). These favorable features have rendered *Halomonas* spp. a next-generation industrial biotechnology platform for various bioproducts, especially poly-hydroxyalkanoates (PHAs) (Lan et al. 2016).

PHAs are microbial polyesters usually accumulated under stress conditions such as extreme temperatures, elevated pH, UV exposure, osmotic shocks, and nutritional imbalances in C/N/P (Obruca et al. 2021; García-Torreiro et al. 2016). Importantly, the former compounds can be employed as biodegradable bio-based alternatives for plastics derived from petrochemicals (Moradali and Rehm 2020). In particular, poly-hydroxybutyrate (PHB) production is an active research field due to its similar mechanical qualities to polypropylene and polyethylene (Balaji et al. 2013; McAdam et al. 2020). Current PHB bioproduction costs are 3 to 4 times higher than traditional fossil fuel-derived plastics, which has precluded broad adoption of biotechnological production processes (Digregorio 2009). While many native microorganisms can produce PHB, only a subset accumulates large amounts that make them suitable for industrial bioproduction. This group includes *Cupriavidus necator* (up to 90% in cell dry weight Dalsasso et al. 2019), *Alcaligenes latus* (up to 39% cell dry weight Wang et al. 2012), and *Azotobacter vinelandii* (up to 79% cell dry weight Díaz-Barrera et al. 2016). Some microorganisms have been also engineered to produce PHB. Most notably, *E. coli* has been recently shown to accumulate up to 93% of its dry cellular weight in PHB (Zhang et al. 2018).

*H. campaniensis* has been pointed out as a promising microbial cell factory due to its ease of cultivation and relatively high PHB accumulation (Da et al. 2022). Strazzullo et al. (2008) evaluated various media conditions in shake-flask cultures reaching accumulations of up to 10% wet cell weight using glucose and yeast extract as C and N sources, respectively. Subsequent work reported increased PHB accumulation in this organism up to 57% and 19% dry cell weight when employing mineral media supplemented with yeast extract and complex media emulating kitchen wastes, respectively (Yue et al. 2014). Other reports have focused their attention to the actual accumulation capabilities. A recent autoflocculating *H. campaniensis* strain reached up to 85% PHB cell dry weight versus 70% of the wild-type non-flocculating strain, when cultured in media with glucose and yeast extract as C and N sources, respectively (Ling et al. 2019). In all the above cases, strategies have been intuitively proposed and empirically evaluated, yielding little insight about other possible limitations for PHB production in this bacterium (Mitra et al. 2020). Altogether, the biotechnological potential of *Halomonas*, their metabolic capabilities, limitations, and PHB production potential remains poorly understood and to a great extent unknown.

In order to better understand and improve PHB production in *Halomonas* spp., we constructed a detailed genome-scale network reconstruction (GENRE) of *H. campaniensis* metabolism, HaloGEM, a moderately halophilic species reported to accumulate up to 70% PHB in dry cell weight basis (Romano et al. 2005; Yue et al. 2014; Ling et al. 2019). A systematic network reconstruction workflow supported by literature information and guided by a pan-genome analysis was employed to build a high-quality genome-scale metabolic model (GEM) (McCubbin et al. 2020), thereby enabling integration of experimental data and interrogation of its metabolic capabilities using constraint-based modelling methods under carbon-sufficient, nitrogen-limited conditions. Computational simulations with HaloGEM predicted candidate nitrogen source mixtures for increasing biomass yield and PHB titer, which were experimentally validated leading to more optimal culture media for PHB production. Furthermore, HaloGEM suggested metabolic engineering interventions for increasing PHB yield, which can guide future efforts for improving its potential as a next-generation microbial platform for bioplastics production.

## Materials and methods

### Metabolic network reconstruction

MetaDraft (Olivier et al. 2020) was employed for the automated generation of initial draft GENREs using the annotated genomes from *H. campaniensis* and four other *Halomonas* spp. available at the NCBI database (Supplementary Information Table S1). A common problem that arises during automatic reconstruction is the incorrect or missing annotation of metabolic genes. To tackle this obstacle, MetaDraft uses previously built high-quality GEMs as templates for metabolic network reconstruction (Olivier et al. 2020). For this task, a GEM from *Chromohalobacter salexigens* (Piubeli et al. 2018), a member of the *Halomonacedeae* family, was chosen as a template. This model was chosen since this species is phylogenetically close, and more importantly, the former has a higher quality than other model alternatives, as demonstrated by the increased number of mapped reactions and compounds as well as metabolic identifiers employed (Supplementary Information Table S6). Network gaps were then automatically filled to generate a functional model as described in Kumar et al. (2007). Briefly, only reactions present in the template model were considered as candidates for bridging network gaps that enabled cellular growth *in silico* under known experimental conditions. The latter avoided the inclusion of spurious reactions. The details for this automated gap-filling can be found in the Supplementary Information Text S1.1.

Following the initial network reconstruction process, a sequence of additional manual refinement steps was undertaken. First, a pan-genome guided strategy (McCubbin et al. 2020) was implemented whereby the previous reconstruction process was replicated for four close *Halomonas* species, namely: *H. boliviensis* LC1, and *Halomonas spp.* strains ALS9, ISL56 and ISL104 (Mandakovic et al. 2020). These reconstructions served as an additional source of information accounting for reactions that could have been initially lost in the reconstruction and hold biological support. A pairwise comparison of these additional reconstructions against *H. campaniensis* yielded putative reactions that were verified in the literature and manually incorporated if appropriate. Additionally, unique reactions of *H. campaniensis* were determined using RAVEN Toolbox, a template-free reconstruction tool (Wang et al. 2018). By integrating available data from KEGG and MetaCyc, RAVEN enabled the generation of additional draft GENREs for both *C. salexigens* and *H. campaniensis* for the identification of unique missing reactions. These reactions were manually checked and incorporated to the metabolic reconstruction if appropriate. Throughout this step, gene-protein-reaction associations were incorporated into the reconstruction based on the reported information.

The final phase entailed a comprehensive manual curation step. Briefly, an exhaustive revision for the candidate and missing reactions based on experimental evidence was performed, e.g., PHB production and alternative carbon source utilization pathways (Kucera et al. 2018). Reactions and metabolites were also checked and balanced for mass and charge according to the physico-chemical characteristics of the modelled compartments. GEMs are typically built simulating neutral pH, i.e., close to 7 (Thiele and Palsson 2010), which is inappropriate in this case for extracellular metabolites. Since *H. campaniensis* is an alkaliphile bacterium that grows at a pH close to 9, there is a widespread use of proton pumps to maintain homeostatic intracellular conditions, e.g., pH close to 7 (Tripathi et al. 2021). Incorrect description of metabolite charges could affect the chemiosmotic gradient, which could lead to an improper functioning of the ATP synthase (Padan et al. 2005). Hence, the charges of extracellular metabolites were revised based on their pKas and corrected if needed. Once all extracellular metabolites were checked, the reactions involving the latter were again revised for mass and charge consistency. Finally, the resulting reconstruction was evaluated using the MEtabolic MOdel TEst suite (MEMOTE, Lieven et al. 2020) for assessing its overall quality and compliance with community standards. The report can be accessed at https://github.com/SysBioengLab/HaloGEM/tree/main/memote_report.

### Strains and culture media

*H. campaniensis* strain AG5 was purchased from ATCC (BAA-966). Seeds were activated in a modified LB medium containing 60 g/L of sodium chloride (LB60). For PHB production, the seed production medium (MM-G) from Tan et al. (2011) was employed supplemented with 30 g/L of glucose, 60 g/L of sodium chloride, and pH adjusted to 9.0 using 2.5 M NaOH. Yeast extract was replaced from MM-G as it hindered proper modeling of cellular growth. Instead, glutamate and ammonium were used as nitrogen sources based on previous observations (Quillaguamán et al. 2008). Minimal media were designed and evaluated using the same amount of nitrogen in the form of only glutamate (2.75 g/L), or, an equimolar mixture of glutamate (1.38 g/L) and $\text {NH}_{\text {4}} \text {Cl}$ (0.5 g/L) (18.7 mmol/L). Biotin was also supplemented (0.05 mg/L) to avoid nutritional limitations (Strazzullo et al. 2008). As initial experimental results suggested nitrogen-limited growth for *H. campaniensis*, different culture media formulations were later computationally predicted and experimentally evaluated. To enable a fair comparison between formulations, the nitrogen was normalized to have the same nitrogen molar concentration (18.7 mmol/L) as the initial cultures. The detailed culture medium composition can be found in the Supplementary Information Text 1.2, and Tables S3, S4 and S5.

### Shake-flask cultivations

Pre-seed (activation) cultures were prepared in 250 mL shake-flasks using 50 mL of LB60 medium and were cultured for 12–16 h. Seed cultures were prepared from the latter cultures in 50 mL of the evaluated medium in 250 mL shake-flasks for 18–24 h. Seed cultures served as starting inoculum for production cultures. The latter were prepared in 1-L shake flasks with 300 mL of the evaluated medium for 70–84 h. All cultures were grown at 37 °C and 200 rpm. In each passaging, cultures were centrifuged at 6000 g for 10 min and resuspended in fresh medium to remove residual media components and inoculated to an initial optical density ($\text {OD}_{\text {600}}$) of 0.2. All experiments were performed in independent biological duplicates.

### Analytical methods

Dry cell biomass was obtained as follows: samples of 1.5–1.8 mL of well-mixed culture were added to previously weighed 2-mL tubes and centrifuged at 10,000 g for 15 min. The supernatant was stored for the analysis of extracellular metabolites, whereas the pellet was resuspended with 1 mL of distilled water and centrifuged again at 10,000 g for 15 min. The supernatant was discarded and tubes were dried at 37 °C until constant mass was reached (approximately 24 h). In the case of extracellular metabolites, glucose, ammonium, and glutamate were measured from the culture supernatant using an Analyzer Y15 (Biosystems, Barcelona, Spain) following the manufacturer’s instructions.

PHB was quantified using the crotonic acid method (Díaz-Barrera et al. 2016). Briefly, 3 mg of dry cell biomass were treated with 1 mL of $\text {H}_{\text {2}}\text {SO}_{\text {4}}$ and incubated at 90 °C and 700 rpm for 1 h. Samples were diluted 15 times, filtered through a 0.22 μm PVDF filter, and assayed through a HPLC-UV system (LC-4000 series, Jasco, Japan) with an Aminex HPX-87 H ion-exclusion column.

### Specific consumption and production rates

The specific secretion and uptake rates for key metabolites were determined from batch experiments using $r_i = \mu /Y_{i,x}$, where μ is the specific growth rate and $Y_{i,x}$ denotes the biomass yield on metabolite *i*. μ was estimated in the exponential growth phase as the slope of the natural logarithm of biomass concentration versus time. Growth data from the lag phase was ignored for these calculations. On the other hand, $Y_{i,x}$ was calculated as the slope of the respective metabolite concentration versus biomass concentration in the exponential growth phase. See Supplementary Information Text S1.2 for more details.

### Flux simulations and predictions evaluation

Flux Balance Analysis (FBA) (Orth et al. 2010) was performed using the reconstructed GEM for predicting cellular growth as the biological objective under different experimental conditions (see Supplementary Information Table S2 for base media definition). Briefly, FBA consists of a linear optimization problem where the biological objective (cellular growth) is maximized subject to steady-state mass balance and capacity constraints. A qualitative evaluation of the model capabilities was carried out by assessing the agreement against growth/non-growth and metabolic consumption/production data reported in the literature. In each test, flux exchanges were modified to simulate the corresponding growth condition. A confusion matrix summarizing correct and incorrect model predictions was built using its geometric mean as a measure of accuracy (Loira et al. 2017). FBA simulations were also employed to compute maximum theoretical yields and determine gene essentiality. In the latter case, *in silico* gene knockouts were simulated under specific culture conditions and its impact on the simulated growth was assessed. Finally, general metabolic capabilities were evaluated using Flux Variability Analysis (FVA) (Mahadevan and Schilling 2003), which enables computation of the feasible ranges for relevant exchange reactions (e.g., fermentation products production) under specific growth conditions.

### Phenotypic phase planes

Phenotypic phase planes (PhPP) (Edwards et al. 2002) were estimated to identify metabolic limitations under different growth conditions. Briefly, 200 equidistant points in the range of 0 to 2 $\text {mmol}/(\text {gDCW}\cdot \text {h})$ were considered and used to fix the specific uptake rates of the carbon (glucose) and nitrogen (glutamate or ammonium) sources. A grid of 200×200 specific uptake rate points was then used to predict the maximum specific growth rate and determine the objective sensitivity to each nutrient consumption (i.e., shadow prices). Changes in the shadow prices denote transitions to different growth limitations, which can be then compared to experimental observations.

### *In silico* media optimization

To assess the effect of different nitrogen sources on the biomass yield, a comparative simulation study was performed using combinations of up to three different nitrogen sources chosen from the available 20 amino acids and ammonium ($\text {NH}_\text {4}$). Oxygen and glucose were assumed to be in excess and not limiting, according to previous experimental observations. For each simulated condition, an FBA problem with biomass growth maximization as an objective function was formulated and solved constraining the maximum allowable nitrogen uptake to 1 $\text {mmol}/(\text {gDCW}\cdot \text {h})$ Eq. 1,

1 $$\begin{aligned} \sum _{i = 1}^{n} w_i \cdot |v_{\text {EX},i}| = 1 \end{aligned}$$

where $|v_{\text {EX},i}|$ denotes the absolute flux value of the exchanged compound *i*, and $w_i$ represents the number of moles of N per mole of compound. For instance, 1 mole of $\text {NH}_\text {4}$ contains 1 mole of N (i.e., $w_i$ = 1), whereas arginine has 4 N moles per mole of molecule (i.e., $w_i$ = 4). The inclusion of this constraint forces the model to choose an optimal mix of N sources from the available alternatives, and thus, avoids solutions where only one nitrogen-rich substrate is always chosen. For these calculations, the lower bound of the NGAM reaction (non-growth-associated maintenance energy) was set to zero. In this way, the optimal objective function value represents the maximum (theoretical) biomass yield under different N source combinations.

Finally, computational results were filtered to remove combinations that either prevented growth or were infeasible. Feasible combinations were sorted in descending order according to the predicted maximum biomass yields on the different nitrogen sources. If less than 10% of the total nitrogen consumed was allocated to a nitrogen source, the contribution of the latter was deemed not significant and could be well described by a simpler medium without it. The simplest combinations with the highest yields were preferred and chosen for experimental validation. Lastly, predicted biomass yields were further refined using the experimental data collected under the most promising conditions. To this task, the most sensitive biomass components were optimized until yield predictions for all conditions agreed with the observations. For this task, a genetic algorithm was employed for the determination of the optimal stoichiometry following the methodology of Campodonico et al. (2016).

### Identification of genetic interventions for higher PHB accumulation

The OptForce algorithm (Ranganathan et al. 2010) was employed to identify promising genetic interventions to increase PHB accumulation in *H. campaniensis*. In short, OptForce identifies up- and down-regulations of genes (including knockouts) that increase the production of a metabolite of interest relative to the wild-type strain. For this task, OptForce solves a multitude of Mixed-Integer Linear Programs (MILP) to identify sets of (one or various) reactions (*Force Sets*) that should be genetically manipulated to achieve the metabolic engineering goal. Given the combinatorial nature of the problem, a reduced set of reactions was used as targets for OptForce. The set of candidate reactions was determined using quantitative Flux Coupling Analysis (qFCA) (Tefagh and Boyd 2019), which identifies the coupling nature (full, partial, directional, or non-existing) between reaction pairs. In particular, reactions coupled to PHB production and nitrogen assimilation in *Halomonas* spp. Kindzierski et al. (2017) were chosen. OptForce was run using the reactions target list excluding essential and exchange reactions, and considering a maximum of three genetic interventions.

### Computational implementation

Flux simulations and computational analyses were performed using the routines and scripts from the COBRA Toolbox v.3.0 (Heirendt et al. 2019) running on the MATLAB Environment (Natick, US). Linear and Mixed-Integer Linear programs were solved using Gurobi optimizer (v. 9.5.2) (Gurobi Optimization, LLC 2023). Refining of the biomass reaction for improving yield predictions under different media was performed using the genetic algorithm (GA) from MATLAB. All calculations were performed in a HP Envy laptop with an Intel(R) Core(TM) i5-7200U CPU 2.71 GHz processor with 8.00 GB of RAM. The code and models used in the reconstruction can be found in the GitHub repository https://github.com/SysBioengLab/HaloGEM.

## Results

### Genome-scale metabolic network of *H. campaniensis*: reconstruction, quality evaluation, and prediction capabilities

**Fig. 1:**
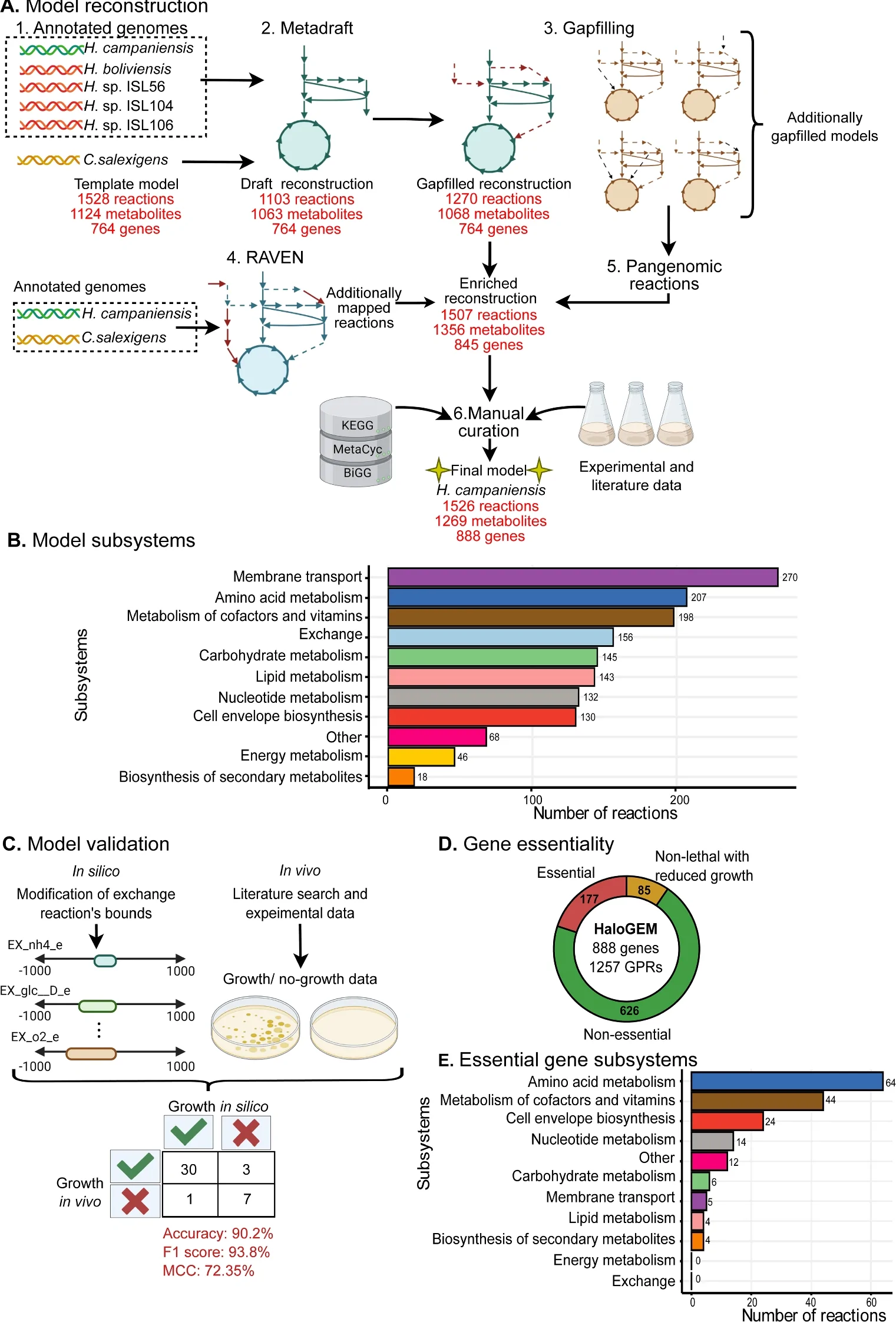
General workflow for the reconstruction and evaluation of predictive capabilities of HaloGEM. **A** Metabolic network reconstruction workflow of HaloGEM. Step 1. Annotated genomes of five *Halomonas* spp. were employed to increase the metabolic coverage and connectivity of the *H. campaniensis* reconstruction following a pan-genome approach. Step 2. MetaDraft was used for the rapid generation of GENREs by using the phylogenetically close organism *C. salexigens* as template. Step 3. Gap-filling was applied to HaloGEM and the additional draft reconstructions using the template model as a source of metabolic reactions. Step 4. RAVEN toolbox was used for *de novo* reconstruction of draft GENREs to identify unique missing reactions not included in the template model. Step 5. An integrative approach was used to incorporate and validate new reactions into the model based on genome evidence from the close draft reconstructions and the results from RAVEN. Step 6. Exhaustive manual curation was performed for the generation of the final model. **B** Reaction subsystems present in the model. **C** Model validation procedure. **D** Gene essentiality analysis. **E** Essential genes subsystems

The initial draft reconstruction for *H. campaniensis* was obtained directly from Metadraft and matched 100% of the genes present in the *i*FP764 model (Fig. 1A step 2). Yet, this model was not functional as it could not simulate growth under minimal media conditions. Gap filling was then employed to improve the metabolic network connectivity. A total of 161 reactions were added with 89.4% corresponding to exchange reactions, 6.2% to transporters, and 4.3% to internal metabolic reactions (Fig. 1A step 3). In parallel, 237 reactions and 290 metabolites were identified by RAVEN toolbox as additional reactions to be added when compared to a reconstruction built *de novo* using *H. campaniensis* and *C. salexigens* genomes (Fig. 1A step 4). Furthermore, to expand the scope of the model, draft reconstructions of close strains *H. boliviensis* LC1, and *Halomonas* spp. strains ISL-56, ISL-104 and ALS9, were built using Metadraft and *i*FP764 as model template. Comparison of the draft reconstructions with the original model yielded 31 candidate metabolic reactions (Fig. 1A step 5). From those, 29 matched previous predictions from RAVEN Toolbox, adding support to the followed workflow. Details about these reactions can be found in the Supplementary Information Table S7.

It is worth noting that the PHB production pathway: PHB synthase (PhaC, reaction ID *PHB_syn_1*), acetoacetyl-CoA reductase (PhaB, reaction IDs *3HBC3E* and *3BTCOAD*), and β-ketothiolase (PhaA, reaction ID *ACACT1r*), had to be manually added as the template reconstruction did not include them (Fig. 1A). For representing the PHB polymer synthesis, we have assumed that four 3-hydroxybutyrate molecules yield a short-chain length PHB (monomer) (McAdam et al. 2020), and 4000 monomers yield a PHB polymer molecule (340 kDa), which lies within the molecular weight range of 340 to 600 kDa reported (Tan et al. 2011). Regardless of the assumed monomer number, it is important to note this choice did not affect PHB yield calculations (Supplementary Information Table S13). Overall, 230 reactions were incorporated to the original reconstruction (Supplementary Information Table S7). The resulting metabolic network reconstruction, HaloGEM, encompassed 1526 reactions (158 exchanges, 297 transport reactions, and 1073 internal metabolic reactions), 1269 metabolites (265 dead-end), and 888 genes (Fig. 1A step 6), with a total of 1257 gene-protein-reaction (GPR) relationships and 151 enzyme complexes. The reconstruction contains 11 subsystems, describing various metabolic functions including central carbon metabolism, nitrogen assimilation and amino acids biosynthetic pathways, membrane transport, cofactors, vitamins, and energy metabolism, and biosynthesis of secondary metabolites. Reactions involved in the synthesis of macromolecular components, e.g., amino acids, carbohydrates, lipids, nucleotides, and cell envelope, constituted most of the reactions in the network (Fig. 1B).

The associated metabolic model was capable of simulating growth and PHB production under various salt concentration conditions, namely: low (0.6 M), medium (1 M), and high salinity (2.5 M). For this task, the biomass reaction equation from *i*FP764 under low and high salt concentrations was employed (Piubeli et al. 2018). The biomass reaction under medium salt concentration (1 M) was estimated by interpolating the stoichiometric coefficients for the main biomass precursor macromolecules and fitting the growth-associated energy requirement (GAM) to experimental data from this study (Supplementary Data Table S20). Finally, evaluation of the reconstruction quality with MEMOTE yielded an overall score of 47%, indicating the presence of 433 blocked reactions, 55 orphan metabolites, and 75 dead-end metabolites. The lack of the Systems Biology Ontology (SBO) annotation explains in large part the relatively low overall score (refer to *Metabolic network reconstruction*). Yet, the model scored highly in stoichiometric consistency (100%) and metabolite connectivity (100%), with high consistency in mass (99.7%) and charge (96.6%) balances. The model also displayed broad annotation coverage of 96.1% and 97% for reaction and metabolite identifiers, respectively. Finally, we note that the template *i*FP764 model did not pass the first model quality check, which prevented a comprehensive comparison against HaloGEM.

To evaluate the predictive power of HaloGEM, cellular growth was simulated *in silico* using FBA under different experimental conditions. In all cases, experimental and simulated biomass production was modelled as a binary variable (growth or no growth) (Fig. 1C). An exhaustive search of previous experimental growth data and experimental results from this study provided 41 growth conditions for simulation evaluation (Supplementary Information Table S8). HaloGEM displayed excellent prediction fidelity, simulating correctly 37 conditions (30 true positives and 7 true negatives) with only three false negatives predicted (L-tyrosine as either a C or N source, and valeric acid as C source) and a single false positive (ammonium as sole N source). Overall, HaloGEM displayed a high performance in this test reaching an accuracy of 90.2%, with a Matthew’s Correlation Coefficient (MCC) of 0.724 and a *F*-score of 93.8%.

Finally, the metabolic capabilities of HaloGEM were further probed using Flux Variability Analysis (FVA) to explore the production of various known fermentation products (Kawata et al. 2016; Ma et al. 2020; Pastor et al. 2013; Ren et al. 2018; Bondar et al. 2022; Hannya et al. 2017; Strazzullo et al. 2008). Overflow products such as acetate, acetaldehyde, citrate, formate, ethanol, and D-lactate could be produced using glucose and glutamate under aerobic conditions as carbon and nitrogen sources, respectively (Supplementary Information Table S15). Additional fermentation products such as ammonium, ectoine, glycolate, glycine-betaine, gluconate, pyruvate, and PHB among others could also be produced (Supplementary Information Table S15). Notably, HaloGEM predicts the obligate efflux of sodium and alanine, and the influx of potassium as recently reported elsewhere (Kindzierski et al. 2017). HaloGEM cannot produce cadaverine. Interestingly, a recent study showed that an engineered *H. campaniensis* was able to produce cadaverine after the integration of this pathway (Zhao et al. 2022). While experimental data on the absence of certain fermentation products is scarce, a comprehensive list of compounds that cannot be produced by HaloGEM is provided for future model verification (Supplementary Information Table S16). In addition, a gene essentiality analysis was performed using the GPR relations encoded in HaloGEM under the previous simulation conditions. From the 888 genes, 177 were found to be essential, 626 non-essential, and 85 non-lethal but when removed they reduced growth (Fig. 1D, Supplementary Information Table S9). As expected, essential genes were mostly associated with key cellular functions such as amino acids, cofactors, and vitamin metabolism, as well as the production of cell envelope components (Fig. 1E). For a full report on the reaction list refer to Supplementary Data Table S21. These results suggest an overall robust behavior of *H. campaniensis* upon genetic perturbations.

**Fig. 2:**
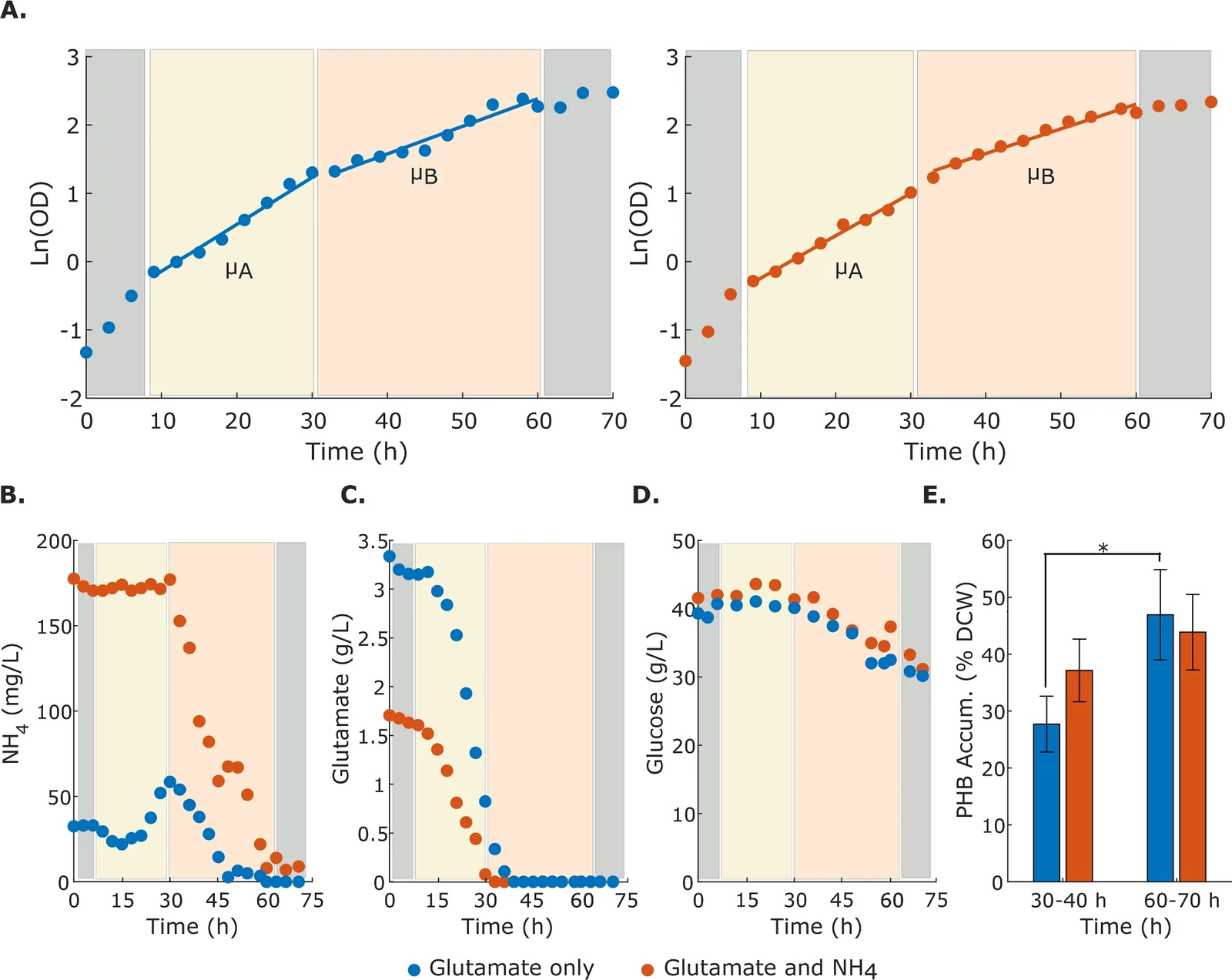
Fermentation kinetics of *H. campaniensis* grown aerobically in minimal media with glucose as C source and glutamate (blue) and glutamate and $\text {NH}_\text {4}$ (orange) as N sources. **A** Growth curves, **B** ammonium, **C** glutamate, **D** glucose, and **E** PHB accumulation profiles. During approximately the first 9 h of fermentation, there is no consumption of the nitrogen sources or glucose (lag phase, in grey). Then, glutamate is consumed and depleted within approx. the first 30 h (growth phase A, shown in yellow). At this point, there is a change in the specific growth rate ($\mu \ (h^{-1}$)), and between 30 and 60 h growth phase B starts (shown in peach). This phase is characterized by the consumption and depletion of ammonium

**Table 1:** Experimental specific consumption and production rates of *H. campaniensis* growing aerobically on minimal media^∗^

Specific rate	Glutamate medium		Glutamate and $\text {NH}_\text {4}$ medium	
	Phase A: 0–30 h	Phase B: 30–70 h	Phase A: 0–30 h	Phase B: 30–70 h
Biomass growth ($\text {1/h}$)	0.073 ± 0.002	0.042 ± 0.006	0.062 ± 0.089	0.039 ± 0.006
Glucose ($\text {mmol/g DCW/h}$)	-0.782 ± 0.25	-0.630 ± 0.153	-0.759 ± 0.141	-0.797 ± 0.24
Glutamate ($\text {mmol/g DCW/h}$)	-1.169 ± 0.014	Not applicable	-0.837 ± 0.083	Not applicable
$\text {NH}_\text {4}$ ($\text {mmol/g DCW/h}$)	0.123 ± 0.153	-0.116 ± 0.018	0.0	-0.077 ± 0.071

^∗^Negative and positive values denote specific uptake and production rates, respectively

**Fig. 3:**
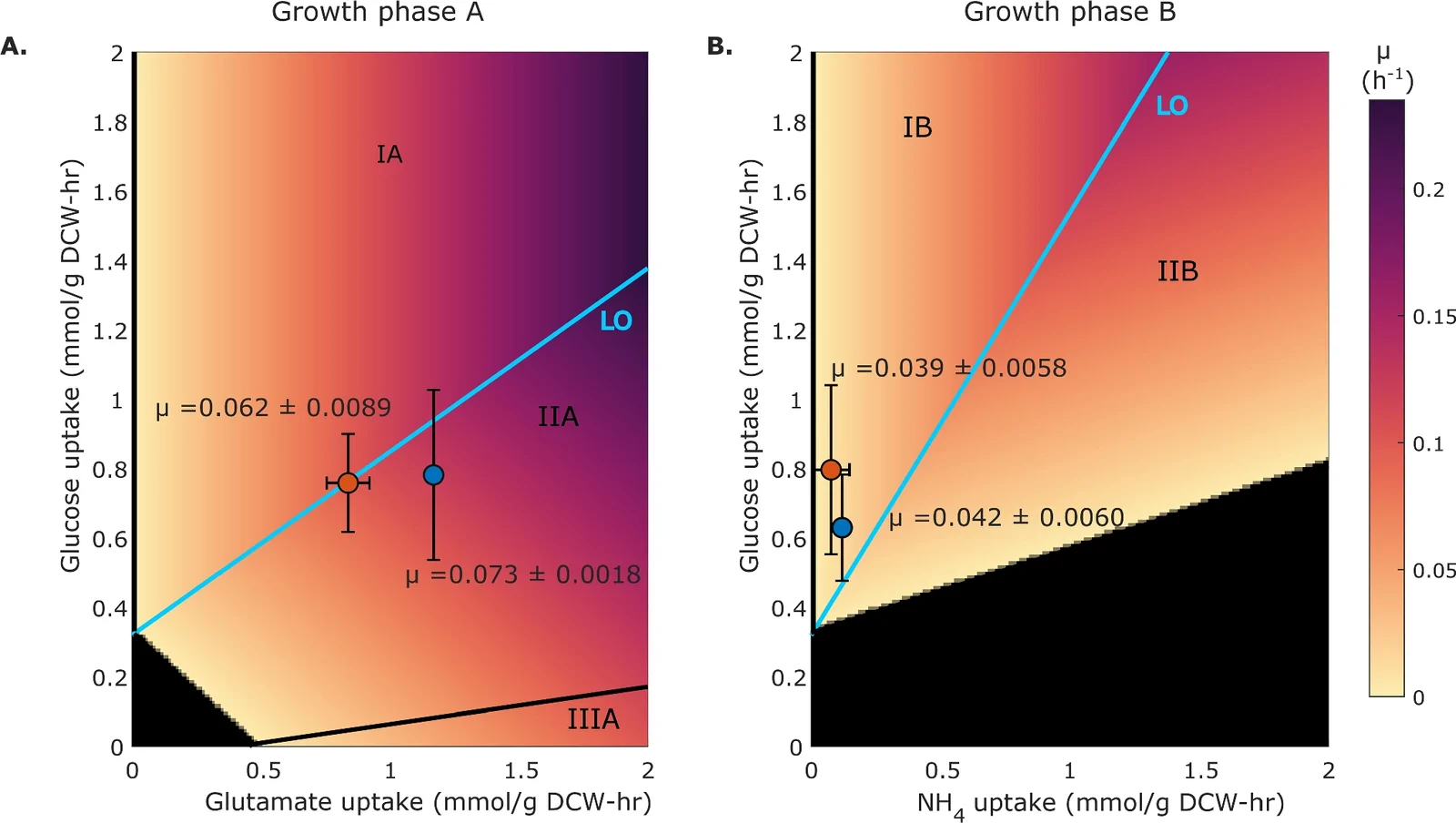
Exploration of the phenotypic landscape of *H. campaniensis* growing aerobically in minimal media using glucose as C source and glutamate or ammonium as N source. **A** PhPP of growth phase A is characterized by the uptake of glutamate and glucose (time 0–30 h, see Fig. 2). Phase IA describes a phenotype where glutamate is limiting and glucose consumption is in excess, whereas phases IIA and IIIA describe phenotypes where glutamate and glucose consumption are in excess but to different proportions. These scenarios are also characterized by distinct product secretion profiles. **B** PhPP of growth phase B is characterized by the uptake of ammonium and glucose (time >30 h, see Fig. 2). Phase IB describes a phenotype where ammonium is limiting and glucose consumption is in excess. Phase IIB corresponds to a phase limited by glucose and with excess $\text {NH}_\text {4}$. In both panels, LO (Line of Optimality in cyan) represents the line that maximizes the biomass yield whereas the black regions denote infeasible phenotypes. The color gradient represents the predicted specific growth rate (μ) for different pairs of substrate uptake rates. Blue and orange dots correspond to growth in medium with glutamate or glutamate and $\text {NH}_\text {4}$ as N source, respectively. Error bars represent two standard deviations around the mean

### Nitrogen source limits aerobic growth of *H. campaniensis* in minimal media

The fermentation growth kinetics of *H. campaniensis* was characterized on minimal media under aerobiosis. Glucose was employed as the main C source, whereas glutamate and a mixture of glutamate and ammonium were employed as N sources (both starting with equal amounts of N). The kinetics of the latter metabolites as well as PHB production, were followed throughout the entire fermentation (approx. 72 h) (Fig. 2). In both conditions, glucose was never depleted and the specific growth rate only slowed down after approx. 60 h of fermentation. This time coincided with the depletion of the nitrogen sources. Interestingly, glutamate and ammonium were not co-consumed, but the former was consumed first and depleted within 30–40 h of fermentation. Ammonium was thereafter consumed, exhibiting two distinct growth phases denoted A and B (Fig. 2B). Cultures with glutamate as the sole N source produced ammonium during the first growth phase (A) at a rate of 0.123 $\text {mmol}/(\text {gDCW}\cdot \text {h})$, which was subsequently consumed at a rate of 0.116 $\text {mmol}/(\text {gDCW}\cdot \text {h})$ after glutamate was exhausted (Table 1, growth phase B). Interestingly, these cultures showed small amounts of ammonium at the beginning of the fermentation, despite not being added to the media. After glutamate depletion, the estimated specific growth rate decreased from 0.073 h^-1^ to 0.042 h^-1^ while the glucose consumption was not significantly affected and remained approximately constant at 0.71 $\text {mmol}/(\text {gDCW}\cdot \text {h})$. A similar pattern was observed in the culture with both glutamate and ammonium (Fig. 2B and C). In this case, glutamate was completely consumed during the first 30 h of fermentation at a rate of 0.837 $\text {mmol}/(\text {gDCW}\cdot \text {h})$, while ammonium concentration remained fairly constant. Then, ammonium was completely consumed at a rate of 0.0077 $\text {mmol}/(\text {gDCW}\cdot \text {h})$. The growth rate also reflected this change, decreasing from 0.062 h^-1^ to 0.039 h^-1^. Similarly, glucose was consumed at a rate of 0.797 $\text {mmol}/(\text {gDCW}\cdot \text {h})$ but not depleted, which is slightly faster than in the previous case. Importantly, the final biomass concentrations were similar in both cases, supporting nitrogen as the limiting nutrient.

In order to gain a deeper insight about the apparent N limitation of *H. campaniensis*, Phenotypic Phase Planes (PhPP) (Edwards et al. 2002) were calculated using specific growth rate maximization as biological objective and the exchanges of glucose and glutamate/ammonium as variables (Fig. 3). The latter were fixed to a range of values within the experimental observations (Table 1). When growing on glutamate as N source (growth phase A), both glutamate and glucose are limiting as described by their corresponding shadow prices and their locations in the PhPP (Fig. 3A, Supplementary Information Table S10). On the other hand, when growing on $\text {NH}_\text {4}$ and glucose (growth phase B, Fig. 3B), experimental points fall within the nitrogen-limited condition as indicated by the null and positive shadow price values of glucose and $\text {NH}_\text {4}$, respectively (Supplementary Information Table S10). Within the experimental measurement error, the model points to a N-limitation underpinning the observed phenotypes under each condition. Finally, experimental points lie fairly close to the optimality line calculated by the model (LO in Fig. 3), supporting flux simulations as a tool for probing the phenotypic landscape of *H. campaniensis* under these conditions.

**Fig. 4:**
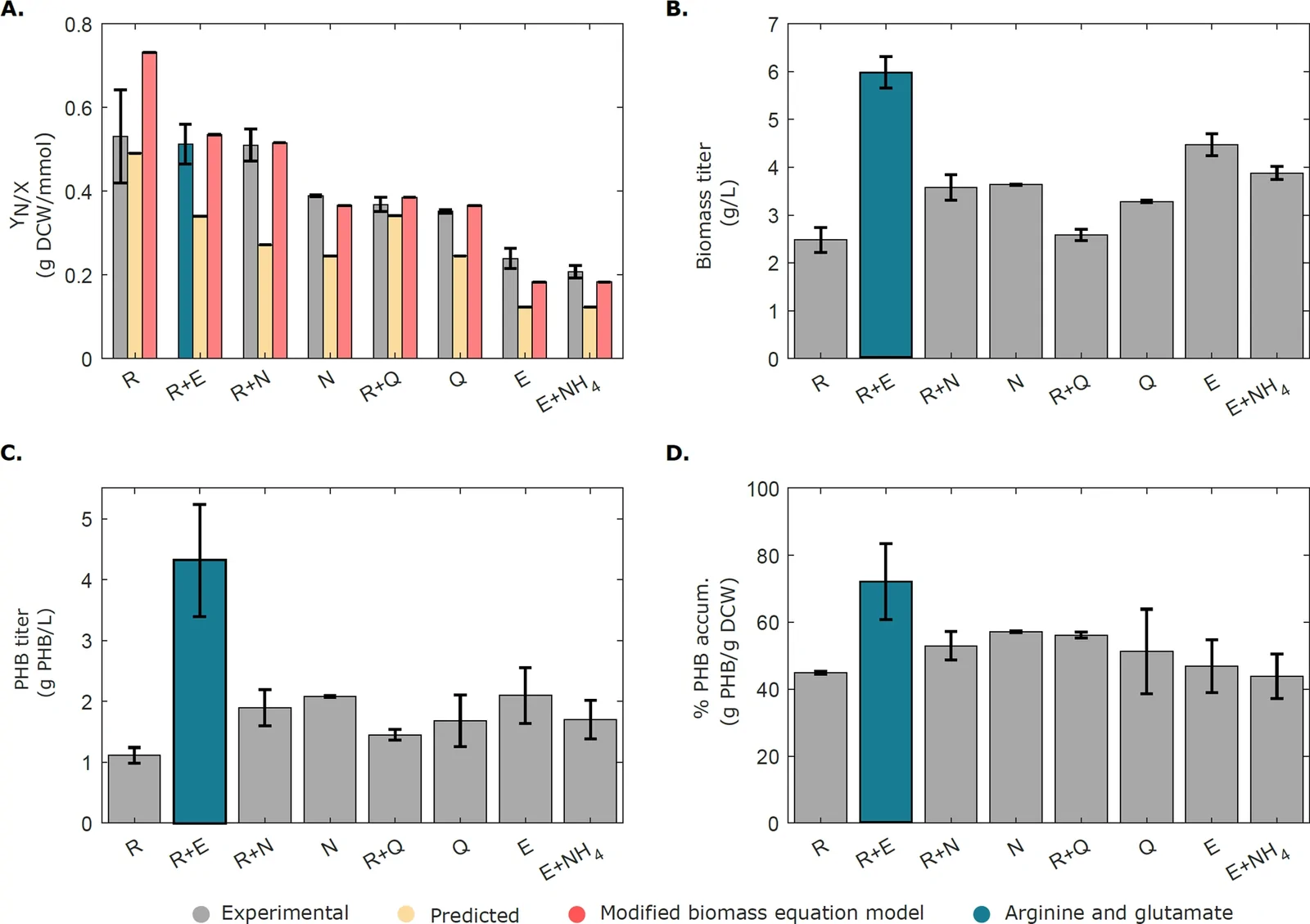
Rational design of fermentation media using HaloGEM for increasing biomass yield and PHB titer. **A** Experimental (grey) and predicted biomass yields by HaloGEM in different N sources using the original (orange) and updated biomass equations (red) (refer to *In silico media optimization*). Experimental biomass titer (**B**), PHB titer (**C**), and PHB accumulation (**D**) under the assessed N sources. **C** PHB titer under the assessed N sources. The best nitrogen source mix is highlighted in blue. Amino acids are represented by their one-letter code, namely: arginine (R), glutamate (E), asparagine (N), and glutamine (Q). Ammonium is represented using its chemical formula $\text {NH}_\text {4}$. Finally, error bars describe two standard deviations around the mean

### Rational media design improves biomass yield and increases PHB titer in *H. campaniensis*

Considering the previous results pointing to N limitation of *H. campaniensis* when growing aerobically on glucose (Fig. 3) and that final PHB accumulation was not substantially different between nitrogen conditions (Fig. 2E), HaloGEM was employed to identify promising N sources that could improve biomass production, and thus, increase PHB titer. More specifically, a total of 1,350 media designs were simulated *in silico* to determine promising nitrogen sources that improve the maximum theoretical biomass yield. Of the simulated media, 145 resulted in zero growth, 120 were infeasible and the remaining 1085 yielded a non-zero result (Supplementary Data Table S19). Following the filtering protocol, 66 unique media were obtained, with arginine ranked at the top (Supplementary Information Table S11). Interestingly, *in silico* flux simulations of media supplemented with arginine and few specific amino acids (e.g., glutamate or glutamine) also predicted high biomass yields. Both glutamate and $\text {NH}_\text {4}$ contribute 1 mole of N per mole, thus, their maximum biomass yields were equal and among the lowest ranked. This result is consistent with previous experimental biomass yield estimations, where no significant change was observed when using only glutamate or a mixture of glutamate/ammonium in the medium (Fig. 2). Notably, in all the simulated scenarios, nitrogen was the limiting nutrient for growth, and more importantly, all the nitrogen source combinations provided the same amount of nitrogen after normalization for yield calculations (Supplementary Information Table S12). While some amino acids were only predicted to be co-consumed in the presence of others (e.g., histidine, isoleucine, leucine, lysine, methionine, phenylanaline, tryptophan, and tyrosine, see Supplementary Data File), other amino acids could individually support growth, namely: arginine, aspartate, asparagine, glutamate, glutamine, glycine, proline, serine and valine. Cysteine and alanine were never consumed in combination with other nitrogen sources (Supplementary Data Table S19).

**Fig. 5:**
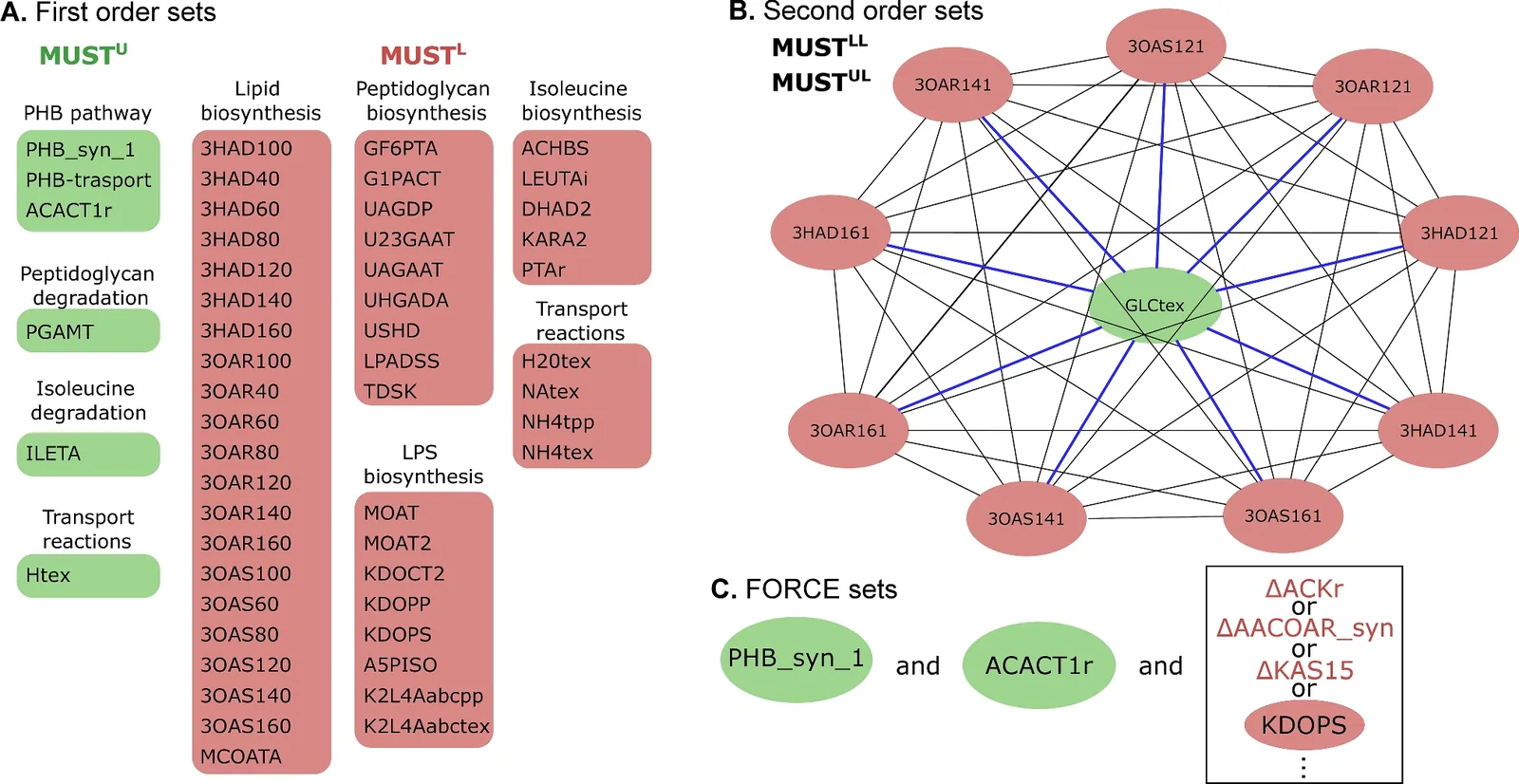
Identification of genetic interventions for improving PHB yield using OptForce. **A** Single-gene interventions identification (First order sets). In green, genes that must increase their expression are grouped in the $MUST^{U}$ set, whereas in red genes that must decrease their expression are shown ($MUST^{L}$). The corresponding subsystem of each gene is also shown in black. **B** Second-order interventions network formed by listing all gene pairs that must be forced to increase PHB yield. Green ovals represent genes that must be up-regulated, whereas red ovals denote genes that must be down-regulated. Each second-order set pair is composed of two connected ovals. **C** Force sets are high-order gene intervention sets that increase PHB yield. These gene combinations represent minimal sets of network changes identified using OptForce. In ovals are represented up- (green) or down-regulation (red), whereas knockouts are depicted by triangles

From the best 15 combinations (Supplementary Information Table S11), 6 media were selected, experimentally tested, and compared against simulation results (Fig. 4A). Media were selected prioritizing the highest biomass yield amino acids as sole nitrogen sources (namely arginine, glutamine, and asparagine), and combinations including arginine as it ranked the highest. In addition, the combination of glutamate and arginine was included to compare the combined effect of glutamate and arginine versus our previous experiments. Notably, all experimental yields were higher than the predicted maximum yields, albeit the latter displayed relatively similar trends between predictions as in the experiments (Fig. 4A in yellow). Maximum yields should only be reachable in theory, and thus, this result conflicts with what should be expected from the model. Given the limited amount of data available and the fact that the biomass reaction should ideally be organism-specific and tailored for each growth condition (Simensen et al. 2022), we estimated a refined general biomass equation that agrees better with experimental observations (Fig. 4A in red). For this task, the most sensitive biomass components were varied until yield predictions for all conditions were closer to the observations (Supplementary Data Table S22). The updated biomass equation showed an 11.7% average relative error, which was approx. 3-fold lower than the original biomass equation (31.5% average relative error). Most importantly, biomass yield predictions now lie within the experimental error for most conditions (Fig. 4A). On the other hand, experimental PHB accumulation remained similar between conditions as assumed with the exception of the best media (arginine and glutamate, Fig. 4C), which showed a 1.64-fold increase in PHB accumulation. Added to the increased biomass titer (Fig. 4B), arginine and glutamate showed an overall improvement in PHB titer of 2.53-fold and PHB productivity of 2.27-fold relative to the initial case. It is worth pointing that the predicted best nitrogen source - arginine -, displayed the highest biomass yield but a low biomass titer.

### *In silico* identification of genetic interventions for increasing PHB accumulation

In order to further improve PHB production, metabolic engineering targets were identified by applying the OptForce algorithm on HaloGEM. Initial results of first order sets, i.e., reactions that should be individually up-regulated, $MUST^{U}$, or, down-regulated, $MUST^{L}$, included reactions from the PHB pathway for up-regulation ($PHB\_syn\_1, PHB\_transport$ and *ACACT*1*r*), and down-regulation of reactions involved in the synthesis of competing products such as lipids, lipopolysaccharide and peptidoglycan (Fig. 5A). Interestingly, reactions associated with isoleucine biosynthesis were identified as targets for down-regulation, suggesting a trade-off between growth and PHB production when using this amino acid.

Second-order sets, which propose the simultaneous intervention of two reactions, i.e., $MUST^{LL}$, $MUST^{UL}$ and $MUST^{UU}$, showed a significant influence of lipid biosynthesis on PHB production. In particular, fatty acids elongation and PHB require acetyl-CoA as a biosynthetic precursor. Down-regulation of reaction pairs $MUST^{LL}$ enabled redirecting more carbon to PHB production (Fig. 5B). This is further supported by the proposed up-regulation of glucose transport coupled to the down-regulation of one of the reactions associated with fatty acids biosynthesis ($MUST^{UL}$ set, Fig. 5B). Using the previous results, three-reaction FORCE sets were computed composed of reactions favoring PHB biosynthesis ($PHB\_syn\_1$ and *ACACT*1*r*) and degradation of its precursors (Fig. 5C). In the case of the competing reactions, the knockouts of $AACOAR\_syn$ and *ACKr* were identified since they drain the PHB precursors (R)-3-Hydroxybutanoyl-CoA and acetyl-phosphate, respectively. On the other hand, *KAS*15 denotes the beta-ketoacyl-ACP synthase, a key enzyme for fatty acid elongation. Similarly, *KDOPS* is associated with lipopolysaccharide (LPS) biosynthesis, and thus, its down-regulation can help to redirect more carbon to PHB (Fig. 5C).

## Discussion

This work presents the first high-quality genome-scale metabolic reconstruction of the haloalkalophilic bacterium *H. campaniensis*. This microorganism is a promising microbial platform for the production of PHB among various other chemicals (Lan et al. 2016; Kawata et al. 2016; Ma et al. 2020; Li et al. 2016). Recent reconstruction efforts for members of the *Halomonas* genus have included *H. syrmensis* (Diken et al. 2015) and two *Halomonas* isolates from industrial brine (Carlson et al. 2016). In both cases, the scope of the metabolic reconstructions was reduced and focused respectively on exopolysaccharides (EPS) secretion (Diken et al. 2015) — which is not the case of *H. campaniensis* (Romano et al. 2005) — and simulation of general metabolic strategies for coping with high salinity and nutrient availability. In contrast, HaloGEM encompasses the entire repertoire of metabolic functions encoded in the genome of *H. campaniensis*, enabling a more holistic and systematic analysis of its capabilities. The predictive power of HaloGEM was reasonably high (Fig. 1C), displaying high accuracy (>90%) for the prediction of growth/no growth under various conditions as tested in other similar organisms (Loira et al. 2017). Notably, carbon source utilization predictions yielded no false positives, illustrating the benefits of following a pan-genome-guided workflow for network curation and gap-filling. Incorrect predictions involved the substrates ammonium and L-tyrosine (nitrogen sources), and valeric acid (carbon source). In the latter case, there are knowledge gaps in the assimilation pathway of *H. campaniensis* for this compound (Lemechko et al. 2019), which explains the model result. The case of L-tyrosine denotes a connectivity problem in the model as only two reactions utilize L-tyrosine (aromatic aminotransferase, TYRTA, and tyrosine-lyase, TYRL), which are blocked and thus cannot redirect L-tyrosine to carbon utilization pathways as suggested experimentally (Strazzullo et al. 2008). Lastly, the ammonium case is more complex. Previous reports indicate that *H. campaniensis* can grow on minimal media with $(\text {NH}_\text {4}) _{\text {2}}\text {SO}_\text {4}$ (Strazzullo et al. 2008) as sole nitrogen source. These results are also partially supported in our experiments when glutamate is depleted (Fig. 2B). However, cultures initiated with ammonium as the sole nitrogen source did not grow (Supplementary Information Figure S1), suggesting a more complex nitrogen assimilation mechanism, possibly concentration-dependent, as recently reported for model organisms like *E. coli* (Kim et al. 2012). As such, further investigation is needed to reveal the underpinning mechanism of ammonium assimilation in *Halomonas*.

Experimental and modeling results point to N as the limiting nutrient during *H. campaniensis* fermentation growing in minimal media with glucose as C source under aerobic conditions. Two media supplemented with the same amount of N but with different sources (glutamate and a mix of glutamate/ammonium) were evaluated based on media employed for PHB production (Tan et al. 2011; Quillaguamán et al. 2008; Strazzullo et al. 2008). Glutamate was preferentially consumed over ammonium while glucose was consumed at an almost constant rate regardless of the N source (Fig. 2D). Throughout the fermentation, glucose was never depleted pointing to N as the limiting nutrient (Fig. 2D). This finding was also supported by the fact that fermentation under both conditions reached similar final biomass and glucose concentrations (Fig. 2A and D). Nitrogen limitation was further supported by an analysis of the biomass growth optimality landscape when varying glucose and glutamate/ammonium uptake rates (Fig. 3). Under either scenario, the N source uptake displayed a significant - if not the greatest - influence on biomass growth as measured by the corresponding shadow price (Fig. 3, Supplementary Information Table S10). Collectively, these results support the application of combined approaches based on models and experimentation for unveiling nutritional limitations.

Nitrogen-limited production of PHB has been previously reported for *Halomonas* (Rivera-Terceros et al. 2015; Ortiz-Veizán et al. 2020) and agrees well with our observations. Yet, there is a lack of systematic studies evaluating the impact of different N sources for improving PHB production in *H. campaniensis*. A previous experimental study reported that ammonium supplemented with either aspartic acid, glycine, glutamine, or glutamate could replace yeast extract in *Halomonas boliviensis* and increase total biomass (Quillaguamán et al. 2008). However, the combinatorial nature of the media design problem discourages an exhaustive experimental screening approach. For this task, *in silico* flux simulations are ideal prospective tools as they enable rapid computation of promising media designs for experimental evaluation. Here, different nitrogen source combinations were simulated *in silico* assuming growth maximization under nitrogen limitation. Computational predictions of media with up to three different N sources yielded satisfactory results and enabled improving biomass yield and PHB titer by 54.2% and 153.4%, respectively, in one of the simulated conditions (arginine and glutamate, Fig. 4A). Results suggest different metabolic assimilation of the various nitrogen sources as indicated by the distinct glucose uptake rates in each scenario (Supplementary Information Table S14). Particularly arginine - the nitrogen source with the highest biomass yield according to the model and our experimental results (Fig. 4A), has been recently reported to boost growth in other bacteria (Valgepea et al. 2017) due to its role in various ATP-related processes (Abdelal 1979). In addition, the most promising media considered various amino acid sources linked directly to TCA intermediates (e.g., alpha-ketoglutarate and oxalacetate), which may explain their positive impact on the biomass yield and PHB production by fueling the TCA cycle and replenishing its intermediates. The case of arginine is notable as it was predicted to have the highest biomass yield but reached one of the lowest biomass titers (Fig. 4B). Interestingly, supplementation of the latter with glutamate yielded the best medium. Glutamate has been reported to play various biological roles in bacteria, particularly as an osmoprotectant (McParland et al. 2021). Intracellular osmoprotectant accumulation (e.g., glutamate, ectoine, and glycine-betaine, Romano et al. (2005)) has been shown to compete with PHB production under stress conditions in *Halomonas* (Strazzullo et al. 2008; Guzmán et al. 2009). Thus, supplementation of this amino acid may be critical for increasing cell viability and may help to explain the higher biomass titer observed (Fig. 4B). Importantly, these results illustrate synergistic effects of different nutrients that could be promising research directions to improve *Halomonas* cultivation.

Among the various uses of genome-scale metabolic models for metabolic engineering, computational strain design probably ranks at the top (Mishra et al. 2018; Vikromvarasiri et al. 2023; Agren et al. 2013). Application of OptForce recapitulated known successful strategies for improving PHB yield (up-regulation of PHB synthetic pathway, Ji et al. 2023), and most notably, provided novel genetic targets that hold promise. In particular, genetic interventions down-regulating biosynthesis of macromolecules such as lipids, LPS and peptidoglycans were identified (Fig. 5A and B). The latter macromolecules act as drains of acetyl-CoA, and thus, compete with PHB production. For instance, fatty acids have been shown to provide precursors for PHAs synthesis (Darani et al. 2013; Tortajada et al. 2013), suggesting that their synthesis should be antagonistic to PHB production. It is worth mentioning that the down-regulation of the above-mentioned targets should be fine-tuned. Associated metabolic reactions are often essential for growth, and thus, they can severely affect the PHB titer and productivity of this intracellular metabolite. Genetic engineering strategies based on promoter engineering (e.g, T7-like and porin promoters), CRISPR-Cas9 systems and SEVA plasmids are feasible alternatives and have been proposed for implementing similar interventions (Chen et al. 2018; Ye and Chen 2021). Other non-metabolic genetic interventions based on self-flocculating bacteria for increased productivity (Ling et al. 2019), or increased cell size for higher intracellular PHB accumulation (Jiang et al. 2017), can be readily combined with the previously mentioned interventions for developing more economically attractive bioprocesses. To this goal, the presented genome-scale metabolic reconstruction will be a valuable tool for driving metabolic engineering experimentation in *H. campaniensis* and other phylogenetically close *Halomonas*.

## Supplementary Information

Below is the link to the electronic supplementary material.

Supplementary file 1 (xlsx 331 KB)

Supplementary file 2 (pdf 319 KB)

## Data Availability

All data accompanying this research are presented directly in the manuscript and supplementary materials.

## Code availability

The employed codes and metabolic model are freely available at https://github.com/SysBioengLab/HaloGEM.
