# Supplementary material for: Metabolic modeling of Halomonas campaniensis improves polyhydroxybutyrate production under nitrogen limitation
Source: Appl Microbiol Biotechnol. 2024 Apr 25;108:310. doi: 10.1007/s00253-024-13111-8 (PMC11045607; doi:10.1007/s00253-024-13111-8)
Supplement: Supplementary file 1 [file 253_2024_13111_MOESM2_ESM.pdf]

Supplementary Information
Applied Microbiology and Biotechnology
Metabolic modeling of *Halomonas campaniensis* improves polyhydroxybutyrate production under nitrogen limitation

Carolina Deantas-Jahn[1], Sebastián Mendoza[1,2], Cuauhtemoc Licona-Cassani[3,4], Camila Orellana[1], and Pedro A. Saa[1,5,*]

[1]Department of Chemical and Bioprocess Engineering, School of Engineering, Pontificia Universidad Católica de Chile, Santiago, Chile
[2]Amsterdam Institute of Molecular and Life Sciences (AIMMS), Vrije Universiteit Amsterdam, Amsterdam, Netherlands
[3]Núcleo de Innovación de Sistemas Biológicos (NISB), FEMSA Biotechnology Center, Tecnológico de Monterrey, Monterrey, Mexico
[4]Escuela de Ingeniería y Ciencias, Tecnológico de Monterrey, Monterrey, Mexico
[5]Instituto de Ingeniería Matemática y Computacional, Pontificia Universidad Católica de Chile, Santiago, Chile
[*]Corresponding author (pnsaa@uc.cl)

February 20, 2024

Contents

1 Supplementary Text . . . **3**
- 1.1 Gap-filling process . . . 3
- 1.2 Experimental media definition . . . 3
- 1.3 Rate formula derivation . . . 3

2 Supplementary Figures . . . **5**

3 Supplementary Tables . . . **6**

List of Figures

S1 Shake-flasks cultures of *H. campaniensis* in minimal media with glutamate and ammonium as nitrogen sources, both at 18.7 mM. As a control, the base medium did not have any nitrogen sources and was supplemented with 30 g/L glucose as C source. While shake flasks with glutamate exhibited cellular growth, no growth was detected for culture with ammonium. The latter was evidenced when comparing the control and the latter culture. . . . 5

List of Tables

S1 GenBank access codes for thestrains used in this study. . . . 6

S2 Constraints used for qualitative model validation simulating media compositions described in literature (Romano et al., 2005). . . . 7

S3 Main media composition . . . 8

S4 Composition of trace elements solution 1 . . . 9

S5 Composition of trace elements solution 2 . . . 10

S6 Comparison between available metabolic models for the reconstruction of the metabolic network of *H. campaniensis*. . . . 11

S7 Manually added reactions to HaloGEM. . . . 12

S8 Qualitative validation of HaloGEM. . . . 20

S9 Gene essentiality analysis statistics. . . . 21

S10 Exchange shadow prices in different fermentation phases. . . . 22

S11 Nitrogen source ranking. Highlighted rows correspond to tested experimental conditions. 23

S12 Biomass yield on various nitrogen sources using glucose as carbon source. . . . 25

S13 Effect of monomer number on PHB composition, molecular weight and maximum theoretical yields calculated with HaloGEM using glucose as carbon source. . . . 26

S14 Nitrogen to glucose consumption ratios for different nitrogen sources under optimal growth and minimum glucose uptake. . . . 27

S15 Reported fermentation products in *Halomonas* . . . 28

S16 Fermentation products that cannot be produced by HaloGEM under the studied conditions. . . . 29

1 Supplementary Text

1.1 Gap-filling process

Network gaps were automatically filled employing the Gapfill algorithm (Kumar et al., 2007) to generate a functional model using *i*FP764 as reaction database. Consequently, only reactions present in the latter model were considered as candidates for bridging the network gaps that enabled cellular growth *in silico* under reported experimental conditions. This measure avoided inclusion of spurious reactions. The GapFill formulation relies on a binary variable y_j defined as (Equation 1):

$$y_j = \begin{cases} 1 & \text{if reaction } j \text{ from the external database is added to the parent network} \\ 0 & \text{otherwise} \end{cases} \tag{1}$$

Since *i*FP764 is a functional model, a subset of its reactions is likely to produce a functional model. A value of α was defined as the percentage of growth in the *i*FP764 model. An additional constraint was added to the GapFill formulation, considering that the biomass reaction has a value that is a percentage of the template model. This percentage was written as α, with values of 1, 80 and 100% used for the gap-filling process.

The reactions that are present for every α and documented in literature were selected and added to the draft reconstruction using the relevant functions from the COBRA toolbox Heirendt et al., 2019. This step was performed for the five reconstructions built.

1.2 Experimental media definition

A modified LB medium was used for growth and seed culture preparation, containing per liter: NaCl 60, tryptone 10 and yeast extract 5. For PHB production, an MM medium was used, with its composition detailed in tables S3,S4 and S5 adapted from Tan et al., 2011. The pH of the medium was adjusted to 9.0 using 2.5 M NaOH. Trace solutions were filter sterilized and added to the autoclaved media; glucose was autoclaved separately from the rest of the medium components. MM media was modified to obtain a defined media based on observationsQuillaguamán et al., 2008 that yeast extract can be substituted with glutamate. To avoid cofactor depletion, the amount of trace solution 1 and 2 were increased to 15 mL/L and 1.5 mL/L respectively. Biotin was added (0.05 mg/L) to account for possible vitamin consumption in absence of yeast extract Strazzullo et al., 2008. Since nitrogen depletion leads to stress in *H. campaniensis* the effect of mixed nitrogen sources was studied, and a new defined media was formulated with glutamate and NH_4Cl. To compare across culture conditions, the initial total amount of nitrogen moles was kept fixed. Nitrogen limitation induces a stress response, thus, a mixed nitrogen medium was formulated to assess cellular response. To compare results, cultures were normalized by nitrogen moles, thus mixed nitrogen media contained 1.38 g/L glutamate and 0.5 g/L NH_4Cl.

1.3 Rate formula derivation

The specific secretion and uptake rates for key metabolites were determined from shake flask experiments using the growth rate and biomass yield on the corresponding metabolite S. The derivation of the rate formula starts with the general mass balance in the fermentation under the assuption of perfect mixing:

$$\frac{d(S \cdot V)}{dt} = F_{in}S_{in} - F_{out}S - r_sXV \tag{2}$$

Where S corresponds to the substrate concentration, F to the mass flux, X to the biomass concentration, V to the reaction volume and r_s to the specific substrate rate. The subscripts in, out refer to quantities inside or outside of the control volume being analysed. In a shake-flask fermentation, the total volume is constant, thus:

$$V \cdot \frac{dS}{dt} = F_{in}S_{in} - F_{out}S_{out} - r_s XV \tag{3}$$

Since it is a batch fermentation, there is no input or output of flow, thus $F_{in} = F_{out} = 0$, then:

$$V \cdot \frac{dS}{dt} = -r_s XV \tag{4}$$

Dividing by the volume and reorganizing:

$$r_s = -\frac{1}{X} \cdot \frac{dS}{dt} \tag{5}$$

Then, the substrate accumulation and biomass concentration yields the sought uptake rate. For the biomass, the specific growth rate (r_x) corresponds to μ yielding the following balance equation:

$$\frac{dX}{dt} = \mu \cdot X \tag{6}$$

μ is a function of biomass and during the exponential phase is constant. By substituting 6 in 5 we get:

$$r_s = -\frac{dS/dt}{dX/dt} \cdot \mu \tag{7}$$

The observed yield Y_{XS} is then defined as:

$$Y_{XS} = \frac{r_s}{r_x} = -\frac{dS/dt}{dX/dt} \tag{8}$$

The above quantity can be estimated by plotting the substrate versus biomass concentration at the same time points and finding the slope, whereas μ can be calculated as the slope of the natural logarithm of the biomass concentration versus time. Then, the experimental substrate uptake rate can be expressed as:

$$r_s = -Y_{XS} \cdot \mu \tag{9}$$

2 Supplementary Figures

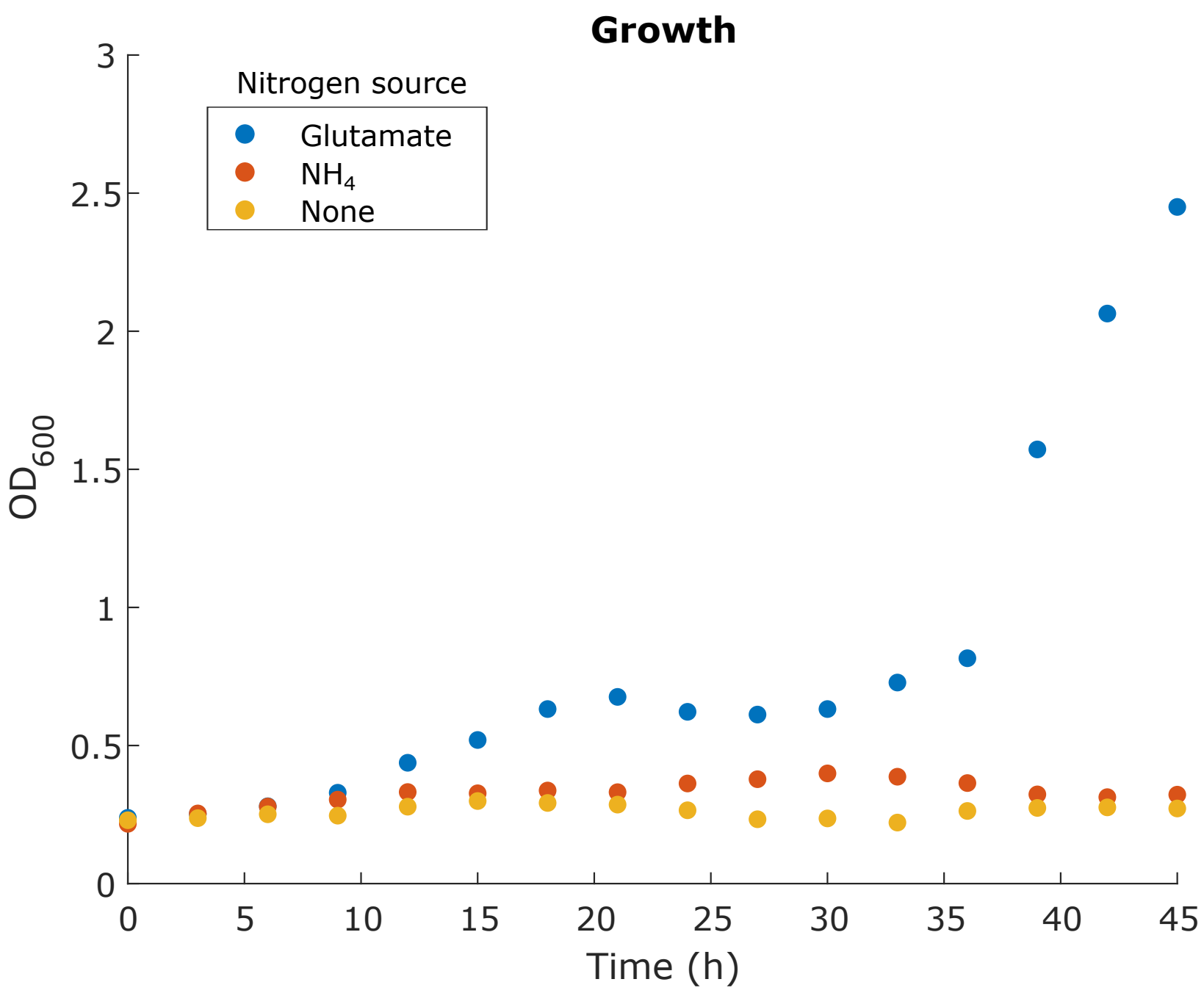

Figure S1: Shake-flasks cultures of *H. campaniensis* in minimal media with glutamate and ammonium as nitrogen sources, both at 18.7 mM. As a control, the base medium did not have any nitrogen sources and was supplemented with 30 g/L glucose as C source. While shake flasks with glutamate exhibited cellular growth, no growth was detected for culture with ammonium. The latter was evidenced when comparing the control and the latter culture.

3 Supplementary Tables

Table S1: GenBank access codes for thestrains used in this study.

Genome	GenBank Assembly Accession	Link
H. campaniensis 5AG	GCA_014193375.1	https://www.ncbi.nlm.nih.gov/assembly/GCF_014193375.1
H. boliviensis LC1	GCA_000236035.1	https://www.ncbi.nlm.nih.gov/assembly/GCF_000236035.1
Halomonas sp. ALS9	GCA_001651035.1	https://www.ncbi.nlm.nih.gov/assembly/GCF_001651035.1
Halomonas sp. ISL104	GCA_018612735.1	https://www.ncbi.nlm.nih.gov/assembly/GCF_018612735.1
Halomonas sp. ISL56	GCA_018612805.1	https://www.ncbi.nlm.nih.gov/assembly/GCF_018612805.1

Table S2: Constraints used for qualitative model validation simulating media compositions described in literature (Romano et al., 2005).

**Reaction ID**	**Value**	**Comments**
EX_k_e	-10	Derived from KH_2PO_4
EX_pi_e	-10	Derived from KH_2PO_4
EX_mg2_e	-10	Derived from $MgSO_4$
EX_so4_e	-10	Derived from $MgSO_4$ and $(NH_4)_2SO_4$
EX_nh4_e	-4	Derived from (NH4)2SO4
EX_na1_e	-10	Derived from NaCl
EX_cl_e	-10	Derived from NaCl
EX_h_e	-10	Always present in media
EX_h2o_e	-10	Always present in media
EX_o2_e	-15	Simulation in aerobic media
EX_ca2_e	-10	Minerals are unconstrained
EX_fe3_e	-10	Minerals are unconstrained
EX_cu2_e	-10	Minerals are unconstrained
EX_zn2_e	-10	Minerals are unconstrained
EX_mn2_e	-10	Minerals are unconstrained
EX_cobalt2_e	-10	Minerals are unconstrained
EX_ni2_e	-10	Minerals are unconstrained
EX_mobd_e	-10	Minerals are unconstrained
EX_btn_e	-0.1	Biotin is added to the medium

Table S3: Culture media compositions.

Component	MM complex media	Defined media	Mixed nitrogen media
Carbon source	30 g/L	30 g/L	30 g/L
NaCl	60 g/L	60 g/L	60 g/L
Yeast extract	1 g/L	-	-
Glutamate	-	2.75 g/L	1.38 g/L
$MgSO_4$	0.2 g/L	0.2 g/L	0.2 g/L
$Na_2HPO_4 \cdot 12H_2O$	9.65 g/L	9.65 g/L	9.65 g/L
KH_2PO_4	1.5 g/L	1.5 g/L	1.5 g/L
NH_4Cl	2 g/L	-	0.5 g/L
Trace element solution 1	10 mL/L	15 mL/L	15 mL/L
Trace element solution 2	1 mL/L	1.5 mL/L	1.5 mL/L
Biotin	-	0.5 mg/L	0.5 mg/L

Table S4: Composition of trace elements solution 1

**Component**	**Composition (g/L)**
Fe(III)–NH_4–citrate	5
$CaCl_2$	2
HCl	1 M

Table S5: Composition of trace elements solution 2

**Component**	**Composition (mg/L)**
$ZnSO_4 \cdot 7H_2O$	100
$MnCl_2 \cdot 4H_2O$	30
H_3BO_3	3300
$CoCl_2 \cdot 6H_2O$	200
$CuSO_4 \cdot 5H_2O$	10
$NiCl_2 \cdot 6H_2O$	20
$NaMoO_4 \cdot 2H_2O$	30
HCl	1 M

Table S6: Comparison between available metabolic models for the reconstruction of the metabolic network of *H. campaniensis*.

**Criteria**	***Chromohalobacter salexigens***	***Halomonas sp. BC1***	***Halomonas smyrnensis***
Number of compounds	1123	309	981
Number of reactions	1528	279	1143
Database identifiers	BiGG	None (arbitrary identifiers)	MetaNetx
Family/Genus	*Halomonacedae / Chromohalobacter*	*Halomonacedae / Halomonas*	*Halomonacedae / Halomonas*
Reference	Piubeli et al., 2018	Carlson et al., 2016	Diken et al., 2015

Table S7: Manually added reactions to HaloGEM.

**Reaction ID**	**Reaction formula**	**Source**
2INSD	1 2ins_c <=>1 h2o_c + 1 dkdi_c	RAVEN
3NUCLE1	1 h2o_c + 1 3amp_c <=>1 pi_c + 1 adn_c	RAVEN
3NUCLE2	1 h2o_c + 1 3ump_c <=>1 pi_c + 1 uri_c	RAVEN
3NUCLE3	1 h2o_c + 1 3cmp_c <=>1 pi_c + 1 cytd_c	RAVEN
3NUCLE4	1 h2o_c + 1 3gmp_c <=>1 pi_c + 1 gsn_c	RAVEN
3OADPCOAT	1 succoa_c + 1 3oxoadp_c <=>1 succ_c + 1 oxadpcoa_c	RAVEN
ACACCT	1 ac_c + 1 aacoa_c <=>1 accoa_c + 1 acac_c	RAVEN
ACKr	1 atp_c + 1 ac_c <=>1 adp_c + 1 actp_c	RAVEN
ADNCYC	1 atp_c <=>1 ppi_c + 1 camp_c	RAVEN
AEPPYRTA	1 pyr_c + 1 2ameph_c <=>1 ala__L_c + 1 Pald_c	RAVEN
ALCD20y	1 nadph_c + 1 h_c + 1 acetone_c <=>1 nadp_c + 1 2ppoh_c	RAVEN
ATHRDHr	1 nadp_c + 1 athr__L_c <=>1 nadph_c + 1 h_c + 1 2aobut_c	RAVEN
CEPA	1 pi_c + 1 cellb_c <=>1 glc__D_c + 1 g1p_c	RAVEN
CODSCL8XI	1 codscl8x_c <=>1 cobya_c	RAVEN
CPC3MT	1 amet_c + 1 copre3_c <=>1 ahcys_c + 1 copre4_c	RAVEN
DHPD	1 h2o_c + 1 56dura_c <=>1 cala_c	RAVEN
DURADx	1 nad_c + 1 56dura_c <=>1 nadh_c + 1 h_c + 1 ura_c	RAVEN
FRDx	1 nad_c + 1 succ_c <=>1 nadh_c + 1 h_c + 1 fum_c	RAVEN
GGGABAH	1 h2o_c + 1 gg4abut_c <=>1 glu__L_c + 1 4abut_c	RAVEN
GUACYC	1 gtp_c <=>1 ppi_c + 1 35cgmp_c	RAVEN
HACD4i	1 nad_c + 1 3hdcoa_c <=>1 nadh_c + 1 h_c + 1 3odcoa_c	RAVEN
HBCO_nadp	1 nadp_c + 1 3hbcoa_c <=>1 nadph_c + 1 h_c + 1 aacoa_c	RAVEN
ICHORS_copy1	1 chor_c <=>1 ichor_c	RAVEN
INS2D	1 nad_c + 1 inost_c <=>1 nadh_c + 1 h_c + 1 2ins_c	RAVEN
IZPN_1	1 h2o_c + 1 4izp_c <=>1 forglu_c	RAVEN
MCDC	1 malcoa_c <=>1 co2_c + 1 accoa_c	RAVEN
MDH	1 nad_c + 1 mal__L_c <=>1 nadh_c + 1 oaa_c + 1 h_c	RAVEN
MSAR	1 nadp_c + 1 3hpp_c <=>1 nadph_c + 1 h_c + 1 msa_c	RAVEN
MTRI	1 5mdr1p_c <=>1 5mdru1p_c	RAVEN
NNDMBRT	1 nicrnt_c + 1 dmbzid_c <=>1 h_c + 1 nac_c + 1 5prdmbz_c	RAVEN
OCOAT1	1 succoa_c + 1 acac_c <=>1 succ_c + 1 aacoa_c	RAVEN
PALDH	1 h2o_c + 1 Pald_c <=>1 pi_c + 1 acald_c	RAVEN
PC20M	1 amet_c + 1 dscl_c <=>1 ahcys_c + 1 h_c + 1 pre3a_c	RAVEN
PC6AR	1 nadp_c + 1 pre6b_c <=>1 nadph_c + 1 h_c + 1 pre6a_c	RAVEN
PRE3BS	1 nadh_c + 1 o2_c + 1 h_c + 1 pre3a_c <=><br>1 h2o_c + 1 nad_c + 1 pre3b_c	RAVEN
PTA2	1 pi_c + 1 ppcoa_c <=>1 coa_c + 1 ppap_c	RAVEN
PTAr	1 pi_c + 1 accoa_c <=>1 coa_c + 1 actp_c	RAVEN
PTRCTA	1 akg_c + 1 ptrc_c <=>1 glu__L_c + 1 4abutn_c	RAVEN
R00150	1 atp_c + 1 nh3_c + 1 h2co3_c <=><br>1 h2o_c + 1 adp_c + 1 cbp_c	RAVEN
R00261	1 glu__L_c <=>1 co2_c + 1 4abut_c	RAVEN
R00282	1 nadph_c + 1 C00028_c + 1 h_c <=>1 nadp_c + 1 C00030_c	RAVEN

Table S7 continued from previous page

R00357	1 h2o_c + 1 o2_c + 1 CE1787_c <=><br>1 nh3_c + 1 h2o2_c + 1 oaa_c	RAVEN
R00481	1 o2_c + 1 CE1787_c <=>1 h2o2_c + 1 iasp_c	RAVEN
R00552	1 h2o_c + 1 arg__L_c <=>1 nh3_c + 1 citr__L_c	RAVEN
R00650	1 amet_c + 1 hcys__L_c <=>1 ahcys_c + 1 met__L_c	RAVEN
R00749	1 etha_c <=>1 nh3_c + 1 acald_c	RAVEN
R01168	1 his__L_c <=>1 nh3_c + 1 urcan_c	RAVEN
R01179	1 ac_c + 1 btcoa_c <=>1 accoa_c + 1 C00246_c	RAVEN
R01196	1 co2_c + 1 accoa_c + 2 h_c + 2 fdxrd_c <=><br>1 coa_c + 1 pyr_c + 2 fdxo_2_2_c	RAVEN
R01353	1 atp_c + 1 C00163_c <=>1 adp_c + 1 ppap_c	RAVEN
R01360	1 C00356_c <=>1 accoa_c + 1 acac_c	RAVEN
R01365	1 btcoa_c + 1 acac_c <=>1 C00246_c + 1 aacoa_c	RAVEN
R01579	1 h2o_c + 1 C00819_c <=>1 nh3_c + 1 glu__D_c	RAVEN
R01625	1 coa_c + 1 C03688_c <=>1 pap_c + 1 ACP_c	RAVEN
R01812	2 nadp_c + 1 thbpt_c <=>2 nadph_c + 2 h_c + 1 C06313_c	RAVEN
R01959	1 h2o_c + 1 Lfmkynr_c <=>1 for_c + 1 Lkynr_c	RAVEN
R02085	1 C00356_c <=>1 h2o_c + 1 3mgcoa_c	RAVEN
R02328	1 g1p_c + 1 dttp_c <=>1 ppi_c + 1 dtdpglu_c	RAVEN
R02466	1 3sala_c <=>1 co2_c + 1 C00519_c	RAVEN
R02487	1 fad_c + 1 glutcoa_c ->1 co2_c + 1 b2coa_c + 1 fadh2_c	RAVEN
R02488	1 glutcoa_c + 1 C04253_c <=><br>1 co2_c + 1 b2coa_c + 1 C04570_c	RAVEN
R02662	1 ibcoa_c + 1 C15973_c <=>1 coa_c + 1 C15977_c	RAVEN
R03055	1 h2o_c + 1 C21028_c <=>1 C21029_c	RAVEN
R03174	1 C15973_c + 1 C15980_c <=>1 coa_c + 1 C15979_c	RAVEN
R03546	1 h_c + 1 h2co3_c + 1 cynt_c <=>1 co2_c + 1 cbm_c	RAVEN
R03877	1 h2o_c + 1 atp_c + 1 mg2_c + 1 ppp9_c <=><br>1 adp_c + 1 pi_c + 2 h_c + 1 mppp9_c	RAVEN
R04097	1 ivcoa_c + 1 C15973_c <=>1 coa_c + 1 C15975_c	RAVEN
R04143	1 atp_c + 1 5mtr_c <=>1 adp_c + 1 5mdr1p_c	RAVEN
R04383	1 5dh4dglcn_c <=>1 C04349_c	RAVEN
R04435	1 pi_c + 1 C06241_c <=>1 h2o_c + 1 pep_c + 1 acmanap_c	RAVEN
R04448	1 atp_c + 1 4mhetz_c <=>1 adp_c + 1 4mpetz_c	RAVEN
R05062	1 C05986_c <=>2 h_c + 2 cytc_c + 1 C05985_c	RAVEN
R05149	2 amet_c + 1 pre6b_c <=><br>1 co2_c + 2 ahcys_c + 1 C06408_c	RAVEN
R05177	1 C06408_c <=>1 hgbyr_c	RAVEN
R05180	1 amet_c + 1 pre3b_c <=>1 ahcys_c + 1 pre4_c	RAVEN
R05181	1 amet_c + 1 pre4_c <=>1 ahcys_c + 1 pre5_c	RAVEN
R05219	1 ahcys_c + 1 ac_c + 1 pre6a_c <=><br>1 h2o_c + 1 amet_c + 1 pre5_c	RAVEN
R05221	1 atp_c + 1 adocbi_c <=>1 adp_c + 1 adocbip_c	RAVEN
R05222	1 gtp_c + 1 adocbip_c <=>1 ppi_c + 1 agdpcbi_c	RAVEN
R05223	1 gmp_c + 1 adocbl_c <=>1 rdmbzi_c + 1 agdpcbi_c	RAVEN

Table S7 continued from previous page

R05225	4 h2o_c + 4 atp_c + 4 gln__L_c + 1 C06506_c <=><br>4 adp_c + 4 pi_c + 4 glu__L_c + 1 adcobhex_c	RAVEN
R05227	1 h2o_c + 1 atp_c + 1 cobalt2_c + 1 hgbam_c <=><br>1 adp_c + 1 pi_c + 1 h_c + 1 co2dam_c	RAVEN
R05285	1 C06753_c <=>2 h_c + 2 cytc_c + 1 C06754_c	RAVEN
R05576	1 nadp_c + 1 C05116_c <=>1 nadph_c + 1 h_c + 1 aacoa_c	RAVEN
R05661	1 atp_c + 1 d5kg_c <=>1 adp_c + 1 d5kgp_c	RAVEN
R05794	1 chol_c + 1 cdpdag_cho_c <=>1 cmp_c + 1 pchol_cho_c	RAVEN
R05808	1 amet_c + 1 C11538_c <=>1 ahcys_c + 1 C17401_c	RAVEN
R05810	1 amet_c + 1 copre4_c <=>1 ahcys_c + 1 codscl5a_c	RAVEN
R05812	1 nadh_c + 1 h_c + 1 copre6_c <=>1 nad_c + 1 codhpre6_c	RAVEN
R06529	1 atp_c + 1 applp_c + 1 adcobhex_c <=><br>1 adp_c + 1 pi_c + 1 adocbip_c	RAVEN
R06530	1 thrp_c <=>1 co2_c + 1 applp_c	RAVEN
R06558	1 gtp_c + 1 adocbi_c <=>1 gdp_c + 1 adocbip_c	RAVEN
R06943	1 fad_c + 1 C14143_c <=>1 fadh2_c + 1 C14144_c	RAVEN
R07229	1 C00030_c + 1 sel_c ->1 h2o_c + 1 C00028_c + 1 slnt_c	RAVEN
R07302	1 atp_c + 1 appl_c + 1 adcobhex_c <=><br>1 adp_c + 1 pi_c + 1 adocbi_c	RAVEN
R07392	1 5mdru1p_c <=>1 h2o_c + 1 2h3k5m_c	RAVEN
R07395	1 h2o_c + 1 2h3k5m_c <=>1 pi_c + 1 dhmtp_c	RAVEN
R07412	1 h2o_c + 2 C00028_c + 1 hemeO_c <=><br>2 C00030_c + 1 hemeA_c	RAVEN
R07599	1 thmpp_c + 1 3mob_c ->1 co2_c + 1 2mhop_c	RAVEN
R07600	1 C15972_c + 1 2mhop_c ->1 thmpp_c + 1 C15977_c	RAVEN
R07601	1 thmpp_c + 1 4mop_c ->1 co2_c + 1 3mhtpp_c	RAVEN
R07602	1 C15972_c + 1 3mhtpp_c ->1 thmpp_c + 1 C15975_c	RAVEN
R07603	1 thmpp_c + 1 3mop_c ->1 co2_c + 1 2mhob_c	RAVEN
R07604	1 C15972_c + 1 2mhob_c ->1 thmpp_c + 1 C15979_c	RAVEN
R07634	1 h2o_c + 1 Lcyst_c <=>1 nh3_c + 1 pyr_c + 1 so3_c	RAVEN
R07772	1 h2o_c + 1 codscl5a_c <=>1 acald_c + 1 codscl5b_c	RAVEN
R07774	1 amet_c + 1 codhpre6_c <=><br>1 co2_c + 1 ahcys_c + 1 codscl7_c	RAVEN
R07775	1 amet_c + 1 codscl7_c <=>1 ahcys_c + 1 codscl8x_c	RAVEN
R07832	1 accoa_c + 1 C16272_c <=>1 ac_c + 1 C16273_c	RAVEN
R08090	1 C04675_c <=>1 ac_c + 1 C16466_c	RAVEN
R08227	1 h2o_c + 1 56dh5flura_c ->1 aflburppa_c	RAVEN
R08503	1 C16737_c <=>1 d5kg_c	RAVEN
R08603	1 h2o_c + 1 dkdi_c <=>1 C16737_c	RAVEN
R08836	1 o2_c + 1 C00355_c <=>1 C17758_c	RAVEN
R09083	1 o2_c + 1 fmnh2_c <=><br>1 h2o_c + 1 e4p_c + 1 dmbzid_c	RAVEN
R09707	1 C19830_c <=>1 C19831_c	RAVEN
R09837	1 C20062_c <=>1 2oxpaccoa_c	RAVEN

Table S7 continued from previous page

R09951	1 nad_c + 1 inost_c <=><br>1 nadh_c + 1 h_c + 1 C20251_c	RAVEN
R09953	1 nad_c + 1 C06153_c <=><br>1 nadh_c + 1 h_c + 1 2ins_c	RAVEN
R09993	1 h2o_c + 1 C20267_c <=>1 nh3_c + 1 4ahmmp_c	RAVEN
R10061	2 amet_c + 1 sarcs_c <=>2 ahcys_c + 1 glyb_c	RAVEN
R10074	1 glutcoa_c + 1 C04253_c <=>1 C02411_c + 1 C04570_c	RAVEN
R10150	1 tet_c + 2 C05359_c <=>2 tsul_c	RAVEN
R10151	5 h2o_c + 1 tsul_c <=>2 so4_c + 10 h_c + 8 cytc_c	RAVEN
R10152	1 undefined_148_c + 1 undefined_158_c <=><br>1 C19692_c + 1 undefined_159_c	RAVEN
R10757	1 dhna_c + 1 C05847_c <=><br>1 co2_c + 1 ppi_c + 1 C19847_c	RAVEN
R10820	1 atp_c + 1 coa_c + 1 3mtp_c <=><br>1 ppi_c + 1 amp_c + 1 C20870_c	RAVEN
R10996	1 thmpp_c + 1 2obut_c <=><br>1 co2_c + 1 C21017_c	RAVEN
R10997	1 C15972_c + 1 C21017_c <=>1 thmpp_c + 1 C21018_c	RAVEN
R10998	1 ppcoa_c + 1 C15973_c <=>1 coa_c + 1 C21018_c	RAVEN
R11026	1 nad_c + 1 C21028_c <=>1 nadh_c + 1 h_c + 1 thym_c	RAVEN
R11264	1 C21250_c <=>1 2mcacn_c	RAVEN
R11555	1 pe_hs_c + 1 lipa_c ->1 dag_hs_c + 1 C21461_c	RAVEN
R11556	1 pe_hs_c + 1 lipa_c <=>1 dag_hs_c + 1 C21462_c	RAVEN
R11557	1 pe_hs_c + 1 C21461_c ->1 dag_hs_c + 1 C21463_c	RAVEN
R11580	1 amet_c + 1 C00030_c + 1 C17401_c <=><br>1 ahcys_c + 1 C00028_c + 1 copre4_c	RAVEN
R12202	1 pe_hs_c + 1 C21994_c <=>1 dag_hs_c + 1 C21995_c	RAVEN
R12644	1 nadp_c + 1 CE2705_c <=><br>1 nadph_c + 1 h_c + 1 C06313_c	RAVEN
R12897	1 atp_c + 1 3mbald_c <=>1 adp_c + 1 C21214_c	RAVEN
RZ5PP	1 h2o_c + 1 5prdmbz_c <=>1 pi_c + 1 rdmbzi_c	RAVEN
SALADC2	1 Lcyst_c <=>1 co2_c + 1 taur_c	RAVEN
TDPDRR	1 nadp_c + 1 dtdprmn_c <=><br>1 nadph_c + 1 h_c + 1 dtdp4d6dm_c	RAVEN
TDPGDH	1 dtdpglu_c <=>1 h2o_c + 1 dtdp4d6dg_c	RAVEN
TMN	1 h2o_c + 1 thm_c <=>1 h_c + 1 4ahmmp_c + 1 4mhetz_c	RAVEN
URCN	1 4izp_c <=>1 h2o_c + 1 urcan_c	RAVEN
r0330	1 nad_c + 1 56dthm_c <=>1 nadh_c + 1 h_c + 1 thym_c	RAVEN
ATPM	h2o_c + atp_c ->h_c + pi_c + adp_c	manual
GLCt2pp	glc__D_p + h_p <=>glc__D_c + h_c	manual
F6Pt6_2pp	2 pi_c + f6p_p <=>f6p_c + 2 pi_p	manual
GLYCtpp	glyc_c <=>glyc_p	manual
PYRt2rpp	h_p + pyr_p <=>h_c + pyr_c	manual
FBP	fdp_c + h2o_c <=>f6p_c + pi_c	manual
ACALDtpp	acald_p <=>acald_c	manual

Table S7 continued from previous page

ACALD	acald_c + coa_c + nad_c <=>accoa_c + h_c + nadh_c	manual
ETOHtrpp	etoh_p <=>etoh_c	manual
PFL	coa_c + pyr_c <=>accoa_c + for_c	manual
FBA3	s17bp_c <=>dhap_c + e4p_c	manual
CITt3pp	h_p + cit_p ->h_c + cit_c	manual
G6Pt6_2pp	2 pi_c + g6p_p ->g6p_c + 2 pi_p	manual
RBK	atp_c + rib__D_c <=>adp_c + h_c + r5p_c	manual
PPK2	atp_c + ppi_c <=>adp_c + pppi_c	manual
ECTtra	ect__L_p <=>ect__L_c	manual
AACPS9	ACP_c + atp_c + octa_c <=>amp_c + ocACP_c + ppi_c	manual
AACPS8	ACP_c + atp_c + dca_c <=>amp_c + dcaACP_c + ppi_c	manual
AACPS7	ACP_c + atp_c + ddca_c <=>amp_c + ddcaACP_c + ppi_c	manual
AACPS6	ACP_c + atp_c + ocdca_c <=>amp_c + ocdcaACP_c + ppi_c	manual
AACPS3	ACP_c + atp_c + hdca_c <=>amp_c + palmACP_c + ppi_c	manual
AACPS1	ACP_c + atp_c + ttdca_c <=>amp_c + myrsACP_c + ppi_c	manual
G5SADs	glu5sa_c <=>1pyr5c_c + h_c + h2o_c	manual
MHPGLUT	hcys__L_c + mhpglu_c <=>met__L_c + hpglu_c	manual
FOLR2	nadph_c + fol_c <=>dhf_c + nadp_c	manual
AACOAT	acac_c + atp_c + coa_c <=>aacoa_c + amp_c + ppi_c	manual
NDPK(dapd:amp)	dadp_c + adp_c <=>camp_c + atp_c + h_c	manual
R02088	dad__2_c + pi_c <=>camp_c + h2o_c	manual
R00706	msa_c + coa_c + nad_c <=>accoa_c + co2_c + nadh_c + h_c	manual
FESD1s	2 h_c + h2o2_c + 2 4fe4s_c ->2 h2o_c + 2 fe3_c + 2 3fe4s_c	manual
FESR	fe2_c + 3fe4s_c ->4fe4s_c	manual
prpB	2mcacn_T_c + h2o_c <=>micit_c	manual
R01623	ACP_c + h2o_c <=>pan4p_c + apoACP_c	manual

Table S7 continued from previous page

BIOMASS_low_salinity	2.6e-05 2fe2s_c + 0.00026 4fe4s_c + 0.0002 5mthf_c + 0.0002 accoa_c + 0.3675 ala__L_c + 0.23851 arg__L_c + 0.07793 asn__L_c + 0.19512 asp__L_c + 59.98 atp_c + 0.0045 ca2_c +0.0045 cl_c + 0.0001 coa_c + 0.003 cobalt2_c + 0.1638 ctp_c + 0.003 cu2_c + 0.03049 cys__L_c + 0.01817 datp_c + 0.032029 dctp_c + 0.03241 dgtp_c + 0.0182 dttp_c + 0.2425 ect__L_c + 0.0002 fad_c + 0.0067 fe2_c + 0.0067 fe3_c + 0.11971 gln__L_c + 0.41808 glu__L_c + 0.25947 gly_c + 0.2086 gtp_c + 45.56 h2o_c + 0.00939 hdect_c + 0.0002 hemeO_c + 0.08145 his__L_c + 0.1459 ile__L_c + 0.1691 k_c + 0.019456 kdo2lipid4_e + 0.3594 leu__L_c + 0.07997 lys__L_c + 0.08023 met__L_c + 0.0075 mg2_c + 0.003 mn2_c + 0.003 mobd_c + 0.013894 murein5px4p_p + 0.263 na1_c + 0.0017 nad_c + 4e-05 nadh_c + 0.0001 nadp_c + 0.0003 nadph_c + 0.0112 nh4_c + 0.0268 pe160_c + 0.045946 pe160_p + 0.0081 pe161_c + 0.02106 pe161_p + 0.10993 phe__L_c + 0.15654 pro__L_c + 0.17139 ser__L_c + 0.0002 sheme_c + 0.0037 so4_c + 9e-05 succoa_c + 0.16669 thr__L_c + 0.04774 trp__L_c + 0.07644 tyr__L_c + 5.5e-05 udcpdp_c + 0.126 utp_c + 0.23021 val__L_c + 0.003 zn2_c ->59.81 adp_c + 59.81 h_c + 58.8062 pi_c + 0.7498 ppi_c	manual

Table S7 continued from previous page

BIOMASS_medium_salinity	2.6e-05 2fe2s_c + 0.00026 4fe4s_c + 0.0002 5mthf_c + 0.0002 accoa_c + 0.333055 ala__L_c + 0.216148 arg__L_c + 0.0706132 asn__L_c + 0.176825 asp__L_c + 56.81 atp_c + 0.0045 ca2_c + 0.0045 cl_c + 0.0001 coa_c + 0.003 cobalt2_c + 0.1612 ctp_c + 0.003 cu2_c + 0.0276294 cys__L_c + 0.0181542 datp_c + 0.0972466 dctp_c + 0.0324077 dgtp_c + 0.0182 dttp_c + 0.338121 ect__L_c + 0.0002 fad_c +0.0067 fe2_c + 0.0067 fe3_c + 0.349719 glu__L_c + 0.235141 gly_c + 0.214914 gtp_c + 56.81 h2o_c + 0.0926154 hdect_c + 0.0002 hemeO_c + 0.0738118 his__L_c + 0.132208 ile__L_c + 0.19456 kdo2lipid4_e + 0.325711 leu__L_c + 0.0724631 lys__L_c + 0.0727073 met__L_c + 0.0075 mg2_c + 0.003 mn2_c + 0.013894 murein5px4p_p + 0.339268 na1_c + 0.0017 nad_c + 4e-05 nadh_c + 0.0001 nadp_c + 0.0003 nadph_c + 0.0112 nh4_c + 0.0268837 pe160_c + 0.045946 pe160_p + 0.00765868 pe161_c + 0.02106 pe161_p + 0.0991598 phe__L_c + 0.141843 pro__L_c + 0.155319 ser__L_c + 0.0002 sheme_c + 0.0037 so4_c + 9e-05 succoa_c + 0.151061 thr__L_c + 0.0432635 trp__L_c + 0.0692726 tyr__L_c + 5.5e-05 udcpdp_c + 0.12309 utp_c + 0.208626 val__L_c + 0.003 zn2_c + 0.108485 gln__L_c ->56.81 adp_c + 56.81 h_c + 56.81 pi_c + 0.745319 ppi_c	manual
BUTt	but_e <=>but_c	manual
IBTt	ibt_e <=>ibt_c	manual
3MBtex	3mb_e <=>3mb_c	manual
PTAt	pta_e <=>pta_c	manual
MALONt	malon_e <=>malon_c	manual
4ABUTt	4abut_e <=>4abut_c	manual
GLUTARt	glutar_e <=>glutar_c	manual
ADPACtd	adpac_e <=>adpac_c	manual
R06944	adpac_c + coa_c + atp_c <=> adpcoa_c + amp_c + ppi_c	manual
R06942	23dhacoa_c + h2o_c <=>3hadpcoa_c	manual
HADPCOADH3	3hadpcoa_c + nad_c <=> h_c + nadh_c + oxadpcoa_c	manual
3OXCOAT	coa_c + oxadpcoa_c <=>accoa_c + succoa_c	manual
OXPTNDH	h2o_c + nad_c + oxptn_c <=> 2 h_c + nadh_c + glutar_c	manual
R12216	glutar_c + akg_c + o2_c <=> 2hglut_c + succ_c + co2_c	manual
APTNAT	akg_c + 5aptn_c <=>glu__L_c + oxptn_c	manual

Table S7 continued from previous page

R12217	2hglut_c + q8_c <=>akg_c + q8h2_c	manual
ABTA	4abut_c + akg_c <=>glu__L_c + sucsal_c	manual
R04002	atp_c + ibt_c <=>adp_c + 2mpylp_c	manual
EATi4	2mpylp_c + coa_c <=>ibcoa_c + pi_c	manual
ACOAD9m	ibcoa_c + fad_c <=>fadh2_c + 2mp2coa_c	manual
R04095	ivcoa_c + fad_c <=>fadh2_c + 3mb2coa_c	manual
R04138	atp_c + 3mb2coa_c + hco3_c <=><br>adp_c + pi_c + 3mgcoa_c	manual
IVCS	atp_c + coa_c + 3mb_c <=>amp_c + ppi_c + ivcoa_c	manual
FACOAL50i	atp_c + coa_c + pta_c <=>amp_c + ppi_c + ptcoa_c	manual
MALCT	malon_c + accoa_c <=>ac_c + malcoa_c	manual
CELLBabc	atp_c + h2o_c + cellb_e <=><br>adp_c + h_c + pi_c + cellb_c	manual
MALT	h2o_c + malt_c <=>2 glc__D_c	manual
MANabcpp	atp_c + h2o_c + man_p <=><br>adp_c + h_c + man_c + pi_c	manual
HEX4	man_c + atp_c ->h_c + adp_c + man6p_c	manual
R04224	2mp2coa_c + h2o_c <=>3hibutcoa_c	manual
3HBCOAHL	3hibutcoa_c + h2o_c <=>coa_c + 3hmp_c	manual
R05066	3hmp_c + nad_c <=>2mop_c + nadh_c + h_c	manual
MMSAD1	coa_c + nad_c + 2mop_c <=><br>co2_c + nadh_c + ppcoa_c	manual
ASNabcpp	h2o_c + atp_c + asn__L_p -><br>asn__L_c + h_c + pi_c + adp_c	manual
3HBC3E	3hbcoa_c <=>3hbcoa__R_c	manual
PHB_syn_1	4 3hbcoa__R_c <=>phb_c + 4 coa_c	manual
PHB-transport	phb_c <=>phb_e	manual
EX_phb_e	phb_e <=>	manual
EX_but_e	but_e <=>	manual
EX_ibt_e	ibt_e <=>	manual
EX_pta_e	pta_e <=>	manual
EX_3mb_e	3mb_e <=>	manual
EX_malon_e	malon_e <=>	manual
EX_4abut_e	4abut_e <=>	manual
EX_glutar_e	glutar_e <=>	manual
EX_adpac_e	adpac_e <=>	manual
EX_gal_e	gal_e <=>	manual
EX_arab__D_e	arab__D_e <=>	manual
EX_cellb_e	cellb_e <=>	manual
EX_xyl__D_e	xyl__D_e <=>	manual

Table S8: Qualitative validation of HaloGEM.

Test Condition	Media	Growth in vivo	Growth in sillico	Result	References
Nutrient essentiality	No Peptone	+	+	TP	Romano et al., 2005
	No Oxygen	-	-	TN	Romano et al., 2005
	No Na	-	-	TN	Romano et al., 2005
Carbon sources	Glucose	+	+	TP	This work; Romano et al., 2005; Yue et al., 2014
	Fructose	+	+	TP	This work; Romano et al., 2005; Yue et al., 2014
	Glycerol	+	+	TP	This work; Romano et al., 2005; Yue et al., 2014
	Sucrose	+	+	TP	This work; Romano et al., 2005; Yue et al., 2014
	Maltose	+	+	TP	This work; Romano et al., 2005
	Xylose	-	-	TN	Romano et al., 2005
	Galactose	-	-	TN	Romano et al., 2005; Yue et al., 2014
	Arabinose	-	-	TN	Romano et al., 2005
	Cellobiose	+	+	TP	Romano et al., 2005; Yue et al., 2014
	Mannose	+	+	TP	Romano et al., 2005; Yue et al., 2014
	Sodium acetate	+	+	TP	Romano et al., 2005; Yue et al., 2014; Strazzullo et al., 2008
	Propionic acid	+	+	TP	Strazzullo et al., 2008
	Butyric acid	+	+	TP	Strazzullo et al., 2008
	Iso-butyric acid	+	+	TP	Strazzullo et al., 2008
	Valeric acid	+	-	FN	Strazzullo et al., 2008
	Iso-valeric acid	+	+	TP	Strazzullo et al., 2008
	Malonic acid	+	+	TP	Strazzullo et al., 2008
	Succinic acid	+	+	TP	Strazzullo et al., 2008
	Glutaric acid	+	+	TP	Strazzullo et al., 2008
	Adipic acid	+	+	TP	Strazzullo et al., 2008
	Citric acid	+	+	TP	Strazzullo et al., 2008
	L-glutamine	+	+	TP	Strazzullo et al., 2008
	Aminobutyric acid	+	+	TP	Strazzullo et al., 2008
	L-asparagine	+	+	TP	Strazzullo et al., 2008
	L-arginine	-	-	TN	Strazzullo et al., 2008
	L-glutamic acid	+	+	TP	Strazzullo et al., 2008
	L-aspartic acid	+	+	TP	Strazzullo et al., 2008
	L-tyrosine	+	-	FN	Strazzullo et al., 2008
	L-proline	+	+	TP	Strazzullo et al., 2008
Both carbon and nitrogen sources, supplemented with NH4	L-glutamine	+	+	TP	Strazzullo et al., 2008
	Amino-butyric acid	+	+	TP	Strazzullo et al., 2008
	L-asparagine	+	+	TP	Strazzullo et al., 2008
	L-arginine	-	-	TN	Strazzullo et al., 2008
	L-glutamic acid	+	+	TP	Strazzullo et al., 2008
	L-aspartic acid	+	+	TP	Strazzullo et al., 2008
	L-tyrosine	+	-	FN	Strazzullo et al., 2008
	L-proline	+	+	TP	Strazzullo et al., 2008
Glucose as a carbon source	Ammonium	-	+	FP	This work.

Table S9: Gene essentiality analysis statistics.

**Category**	**Essential**	**Non-essential**	**Non-lethal with reduced growth**
Amino acid metabolism	64	128	9
Metabolism of cofactors and vitamins	44	87	12
Cell envelope biosynthesis	24	25	0
Nucleotide metabolism	14	26	6
Other	12	86	2
Carbohydrate metabolism	6	105	24
Membrane transport	5	108	4
Biosynthesis of secondary metabolites	4	8	0
Lipid metabolism	4	25	0
Energy metabolism	0	28	28
Exchange	0	0	0
**Total**	**177**	**626**	**85**

Table S10: Exchange shadow prices in different fermentation phases.

**Shadow price**	**Condition A: Glutamate medium**		**Condition B: Glutamate and NH_4 medium**	
	**Phase I**	**Phase II**	**Phase I**	**Phase II**
Glucose	0.0971	0	0.0971	0
Glutamate	0.0671	NA	0.0971	NA
Amonium	NA	0.1174	0	0.1174

Table S11: Nitrogen source ranking. Highlighted rows correspond to tested experimental conditions.

N source 1	N source 2	N source 3	Yield (g DCW/mmol AA)
EX_arg__L_e			0.4910
EX_arg__L_e	EX_gly_e	EX_trp__L_e	0.3493
EX_arg__L_e	EX_lys__L_e	EX_gln__L_e	0.3413
EX_arg__L_e	EX_glu__L_e	EX_phe__L_e	0.3398
EX_arg__L_e	EX_asp__L_e	EX_cys__L_e	0.3398
EX_nh4_e	EX_arg__L_e	EX_ser__L_e	0.3257
EX_arg__L_e	EX_ser__L_e	EX_gln__L_e	0.3108
EX_arg__L_e	EX_his__L_e	EX_asn__L_e	0.2718
EX_arg__L_e	EX_asn__L_e		0.2691
EX_arg__L_e	EX_met__L_e	EX_gln__L_e	0.2665
EX_asn__L_e			0.2455
EX_arg__L_e	EX_asn__L_e	EX_gln__L_e	0.2455
EX_gln__L_e			0.2455
EX_his__L_e	EX_pro__L_e	EX_asn__L_e	0.2219
EX_glu__L_e	EX_met__L_e	EX_asn__L_e	0.2183
EX_lys__L_e	EX_ser__L_e	EX_gln__L_e	0.2179
EX_his__L_e	EX_ser__L_e	EX_asn__L_e	0.2176
EX_glu__L_e	EX_ile__L_e	EX_asn__L_e	0.2169
EX_cys__L_e	EX_gly_e	EX_asn__L_e	0.2159
EX_gly_e	EX_his__L_e	EX_asn__L_e	0.2159
EX_arg__L_e	EX_his__L_e	EX_val__L_e	0.1931
EX_gly_e	EX_pro__L_e	EX_gln__L_e	0.1916
EX_gly_e	EX_val__L_e	EX_gln__L_e	0.1654
EX_nh4_e	EX_cys__L_e	EX_asn__L_e	0.1654
EX_nh4_e	EX_gln__L_e		0.1654
EX_nh4_e	EX_phe__L_e	EX_asn__L_e	0.1654
EX_asp__L_e	EX_pro__L_e	EX_gln__L_e	0.1554
EX_nh4_e	EX_tyr__L_e	EX_gln__L_e	0.1554
EX_asp__L_e	EX_ser__L_e	EX_gln__L_e	0.1554
EX_gly_e	EX_met__L_e	EX_gln__L_e	0.1501
EX_arg__L_e	EX_asp__L_e	EX_his__L_e	0.1444
EX_asp__L_e	EX_his__L_e	EX_gln__L_e	0.1416
EX_nh4_e	EX_his__L_e	EX_gln__L_e	0.1416
EX_arg__L_e	EX_glu__L_e	EX_leu__L_e	0.1414
EX_arg__L_e	EX_glu__L_e	EX_val__L_e	0.1414
EX_arg__L_e	EX_pro__L_e	EX_ser__L_e	0.1334
EX_nh4_e	EX_arg__L_e		0.1334
EX_arg__L_e	EX_ser__L_e		0.1334
EX_nh4_e	EX_his__L_e	EX_pro__L_e	0.1250
EX_his__L_e	EX_pro__L_e	EX_ser__L_e	0.1250
EX_gly_e	EX_his__L_e	EX_pro__L_e	0.1250
EX_asp__L_e	EX_his__L_e	EX_pro__L_e	0.1250
EX_asp__L_e	EX_his__L_e	EX_ser__L_e	0.1250
EX_nh4_e	EX_glu__L_e	EX_lys__L_e	0.1238
EX_asp__L_e	EX_gly_e	EX_lys__L_e	0.1238
EX_pro__L_e	EX_ser__L_e	EX_trp__L_e	0.1234
EX_nh4_e	EX_glu__L_e	EX_phe__L_e	0.1227
EX_asp__L_e	EX_pro__L_e	EX_asn__L_e	0.1227
EX_glu__L_e	EX_ser__L_e	EX_val__L_e	0.1227
EX_gly_e	EX_pro__L_e	EX_ser__L_e	0.1227
EX_asp__L_e			0.1227
EX_asp__L_e	EX_glu__L_e	EX_ser__L_e	0.1227
EX_ser__L_e	EX_val__L_e		0.1227
EX_nh4_e			0.1227
EX_nh4_e	EX_pro__L_e	EX_asn__L_e	0.1227
EX_gly_e			0.1227
EX_asp__L_e	EX_ser__L_e	EX_val__L_e	0.1227
EX_nh4_e	EX_asp__L_e	EX_val__L_e	0.1227
EX_ser__L_e			0.1227

EX_nh4_e	EX_gly_e	EX_pro__L_e	0.1227
EX_asp__L_e	EX_pro__L_e	EX_val__L_e	0.1227
EX_glu__L_e	EX_gly_e		0.1227
EX_val__L_e			0.1227
EX_pro__L_e			0.1227
EX_glu__L_e			0.1227
EX_asp__L_e	EX_cys__L_e	EX_pro__L_e	0.1227

Table S12: Biomass yield on various nitrogen sources using glucose as carbon source.

**Nitrogen source**	**Nitrogen content (mol N/mol N source)**	**Biomass yield (g DCW/mmol N source)**	**Biomass yield (g DCW/mmol N)**
NH4	1	0.1227	0.1227
Arginine	4	0.491	0.1227
Aspartate	1	0.1227	0.1227
Asparagine	2	0.2455	0.1227
Glutamate	1	0.1227	0.1227
Glutamine	2	0.2455	0.1227
Glycine	1	0.1227	0.1227
Proline	1	0.1227	0.1227
Serine	1	0.1227	0.1227

Table S13: Effect of monomer number on PHB composition, molecular weight and maximum theoretical yields calculated with HaloGEM using glucose as carbon source.

Number of monomers	PHB composition	Molecular weight (kDa)	Max. PHB monomer yield (mol/mol)	Max. PHB monomer yield (g/g)
4	$C_{16}H_{24}O_8$	0.344	$3.352 \cdot 10^{-1}$	0.6406
40	$(C_{16}H_{24}O_8)_{10}$	3.44	$3.352 \cdot 10^{-2}$	0.6406
400	$(C_{16}H_{24}O_8)_{100}$	34.4	$3.352 \cdot 10^{-3}$	0.6406
4000	$(C_{16}H_{24}O_8)_{1000}$	344	$3.352 \cdot 10^{-4}$	0.6406
40000	$(C_{16}H_{24}O_8)_{10000}$	3440	$3.352 \cdot 10^{-5}$	0.6406

Table S14: Nitrogen to glucose consumption ratios for different nitrogen sources under optimal growth and minimum glucose uptake.

**Nitrogen source(s)**	**Optimal ratio of nitrogen to glucose uptake (mol/mol)**
Arginine	0.885
Arginine and glutamate	1.514
Arginine and asparagine	0.890
Asparagine	0.886
Arginine and glutamine	1.108
Glutamine	1.007
Glutamate	1.514
Glutamate and ammonium	1.514

Table S15: Reported fermentation products in *Halomonas*

**Species**	**Strain**	**Fermentation products**	**Reference**
H. campaniensis	LC9	cadaverine	Zhao et al., 2022
H. bluephagenesis	TD01	acetate, D-lactate, formate, ethanol	Ren et al., 2018
H. bluephagenesis	TD01	ectoine	Ma et al., 2020
H. bluephagenesis	TD01	L-threonine	Du et al., 2020
H. bluephagenesis	TD01	L-lysine	Zhao et al., 2022
H. boliviensis	DSM 15516	gluconic acid	Bondar et al., 2022
Halomonas sp.	KM1	oxaloacetate, pyruvate	Hannya et al., 2017

Table S16: Fermentation products that cannot be produced by HaloGEM under the studied conditions.

O-Acetyl-L-serine	L-Cysteine	L-Glutamate	lysine	L-Prolinylglycine
3- Methyl butanoic acid	Deoxyguanosine	Glutarate	D-Malate	Pentanoate
4-aminobutanoate	Dihydroxyacetone	Glycine	L-Malate	D-Ribose
Adenine	dIMP	Glycine betaine	Malonate	L-Serine
Adipic acid	Deoxyinosin	Reduced glutathione	Maltose	Succinate
2-Oxoglutarate	Ectoine	GTP	Maltohexaose	D-Mannose 6-phosphate
D-Alanyl-D-alanine	Ethanolamine	Hydrogen sulfide	Maltopentaose	L-Methionine
Allantoin	Formaldehyde	Hexadecanoic-acid	Maltotriose	Nitric oxide
N-Acetyl-D-glucosamine N-Acetylmuramic acid	Fe(III)dicitrate	Hexadecanoic-acid (n-C16:1)	Maltotetraose	Nitrite
D-arabinose	Fe(III)hydroxamate	5-hydroxyectoine	myo-Inositol	Nitrate
aerobactin minus Fe3	Ferrichrome	L-Histidine	KDO(2)-lipid IV A	Sucrose
Aerobactin	Glycerophosphoglycerol	L-Homoserine	L-Lactate	Thymine
L-Arginine	sn-Glycero-3-phospho-1-inositol	Hexanoate (n-C6:0)	L-alanine-D-glutamate	Trehalose
L-Asparagine	Glycerophosphoserine	Glycerol	6-diaminoheptanedioate	L-Tryptophan
L-Aspartate	D-galactose	Glycerol 2-phosphate	D-Mannose	tetradecanoate (n-C14:0)
Biotin	D-Galactonate	Glycerol 3-phosphate	Superoxide anion	tetradecenoate (n-C14:1)
Butyrate	D-Glucosamine 6-phosphate	GMP	octadecanoate (n-C18:0)	L-Tyrosine
D-cellobiose	ferroxamine	Guanosine	octadecenoate	Uracil
Decanoate	D-Fructose	Isobutyrate	octanoate (n-C8:0)	L-Valine
Dodecanoate (n-C12:0)	Fumarate	L-Isoleucine	Orotate	Xanthine
dGMP	sn-Glycero-3-phosphocholine	IMP	L-Phenylalanine	Xanthosine 5-phosphate
Cys-Gly	sn-Glycero-3-phosphoethanolamine	Indole	Propionate	Xanthosine
Choline	GDP	L-alanine-D-glutamate	Propanal	D-xylose
coprogen	D-Glucarate	6-diaminoheptanedioate-D-alanine	Phosphonate	
Cytosine	L-Glutamine	L-Leucine	L-Proline	

References

Bondar, M., Pedro, F., Oliveira, M. C., da Fonseca, M. M. R., & Cesário, M. T. (2022). Red algae industrial residues as a sustainable carbon platform for the co-production of poly-3-hydroxybutyrate and gluconic acid by halomonas boliviensis. Frontiers in Bioengineering and Biotechnology, 10. https://doi.org/10.3389/fbioe.2022.934432

Carlson, R. P., Oshota, O., Shipman, M., Caserta, J. A., Hu, P., Charles, Saunders, W., Xu, J., Jay, Z. J., Reeder, N., Richards, A., Pettigrew, C., Brent, & Peyton, M. (2016). Integrated molecular, physiological and in silico characterization of two *Halomonas* isolates from industrial brine. Extremophiles, 20. https://doi.org/10.1007/s00792-015-0806-6

Diken, E., Ozer, T., Arikan, M., Emrence, Z., Oner, E. T., Ustek, D., & Arga, K. Y. (2015). Genomic analysis reveals the biotechnological and industrial potential of levan producing halophilic extremophile, *Halomonas smyrnensis AAD6T*. SpringerPlus. https://doi.org/10.1186/s40064-015-1184-3

Du, H., Zhao, Y., Wu, F., Ouyang, P., Chen, J., Jiang, X., Ye, J., & Chen, G.-Q. (2020). Engineering halomonas bluephagenesis for l-threonine production. Metabolic Engineering, 60, 119–127. https://doi.org/https://doi.org/10.1016/j.ymben.2020.04.004

Hannya, A., Nishimura, T., Matsushita, I., Tsubota, J., & Kawata, Y. (2017). Efficient production and secretion of oxaloacetate from halomonas sp. km-1 under aerobic conditions. AMB Express, 7(1), 209. https://doi.org/10.1186/s13568-017-0516-9

Heirendt, L., Arreckx, S., Pfau, T., Mendoza, S. N., Richelle, A., Heinken, A., Haraldsdóttir, H. S., Wachowiak, J., Keating, S. M., Vlasov, V., Magnusdóttir, S., Ng, C. Y., Preciat, G., Žagare, A., Chan, S. H. J., Aurich, M. K., Clancy, C. M., Modamio, J., Sauls, J. T., Noronha, A., Bordbar, A., Cousins, B., Assal, D. C. E., Valcarcel, L. V., Apaolaza, I., Ghaderi, S., Ahookhosh, M., Guebila, M. B., Kostromins, A., Sompairac, N., Le, H. M., Ma, D., Sun, Y., Wang, L., Yurkovich, J. T., Oliveira, M. A. P., Vuong, P. T., Assal, L. P. E., Kuperstein, I., Zinovyev, A., Hinton, H. S., Bryant, W. A., Artacho, F. J. A., Planes, F. J., Stalidzans, E., Maass, A., Vempala, S., Hucka, M., Saunders, M. A., Maranas, C. D., Lewis, N. E., Sauter, T., Palsson, B. Ø., Thiele, I., & Fleming, R. M. T. (2019). Creation and analysis of biochemical constraint-based models using the cobra toolbox v.3.0. Nat. Protoc., 6, 1290–1307. https://doi.org/10.1038/nprot.2007.99

Kumar, V. S., Dasika, M. S., & Maranas, C. D. (2007). Optimization based automated curation of metabolic reconstructions. BMC Bioinform., 8. https://doi.org/10.1186/1471-2105-8-212

Ma, H., Zhao, Y., Huang, W., Zhang, L., Wu, F., Ye, J., & Chen, G.-Q. (2020). Rational flux-tuning of halomonas bluephagenesis for co-production of bioplastic phb and ectoine. Nature Communications, 11(1), 3313. https://doi.org/10.1038/s41467-020-17223-3

Piubeli, F., Salvador, M., Argandoña, M., Nieto, J. J., Bernal, V., Pastor, J. M., Cánovas, M., & Vargas, C. (2018). Insights into metabolic osmoadaptation of the ectoines-producer bacterium *Chromohalobacter salexigens* through a high-quality genome scale metabolic model. Microb. Cell Factories, 17, 1–20. https://doi.org/10.1186/S12934-017-0852-0

Quillaguamán, J., Doan-Van, T., Guzman, H., Guzman, D., Martin, J., Everest, A., & Hatti-Kaul, R. (2008). Poly(3-hydroxybutyrate) production by *Halomonas boliviensis* in fed-batch culture. Appl. Microbiol. Biotechnol., 78, 227–232. https://doi.org/10.1007/s00253-007-1297-x

Ren, Y., Ling, C., Hajnal, I., Wu, Q., & Chen, G.-Q. (2018). Construction of halomonas bluephagenesis capable of high cell density growth for efficient pha production. Applied Microbiology and Biotechnology, 102(10), 4499–4510. https://doi.org/10.1007/s00253-018-8931-7

Romano, I., Giordano, A., Lama, L., Nicolaus, B., & Gambacorta, A. (2005). *Halomonas campaniensis* sp. nov., a haloalkaliphilic bacterium isolated from a mineral pool of campania region, italy. Syst. Appl. Microbiol., 28, 610–618. https://doi.org/10.1016/j.syapm.2005.03.010

Strazzullo, G., Gambacorta, A., Vella, F. M., Immirzi, B., Romano, I., Calandrelli, V., Nicolaus, B., & Lama, L. (2008). Chemical-physical characterization of polyhydroxyalkanoates recovered by means of a simplified method from cultures of *Halomonas campaniensis*. World J. Microbiol. Biotechnol., 24, 1513–1519. https://doi.org/10.1007/s11274-007-9637-7

Tan, D., Xue, Y. S., Aibaidula, G., & Chen, G. Q. (2011). Unsterile and continuous production of polyhydroxybutyrate by *Halomonas* td01. Bioresour. Technol., 102, 8130–8136. https://doi.org/10.1016/j.biortech.2011.05.068

Yue, H., Ling, C., Yang, T., Chen, X., Chen, Y., Deng, H., Wu, Q., Chen, J., & Chen, G.-Q. (2014). A seawater-based open and continuous process for polyhydroxyalkanoates production by recombinant *Halomonas campaniensis* ls21 grown in mixed substrates. Biotechnol. Biofuels, 7, 1–12. https://doi.org/10.1186/1754-6834-7-108

Zhao, C., Zheng, T., Feng, Y., Wang, X., Zhang, L., Hu, Q., Chen, J., Wu, F., & Chen, G.-Q. (2022). Engineered halomonas spp. for production of l-lysine and cadaverine. Bioresource Technology, 349, 126865. https://doi.org/https://doi.org/10.1016/j.biortech.2022.126865
